# Circulating small extracellular vesicle-derived splicing factor 3b subunit 4 as a non-invasive diagnostic biomarker of early hepatocellular carcinoma

**DOI:** 10.1186/s13046-023-02867-y

**Published:** 2023-10-30

**Authors:** Ju A Son, Ji Hyang Weon, Geum Ok Baek, Hye Ri Ahn, Ji Yi Choi, Moon Gyeong Yoon, Hyo Jung Cho, Jae Youn Cheong, Jung Woo Eun, Soon Sun Kim

**Affiliations:** 1 Department of Gastroenterology, Ajou University School of Medicine, Suwon, Republic of Korea; 2 Department of Biomedical Sciences, Ajou University Graduate School of Medicine, Suwon, Republic of Korea

**Keywords:** Biomarker, Myeloid-derived suppressor cell, Liquid biopsy, Liver Neoplasms, SF3B4

## Abstract

**Background:**

Hepatocellular carcinoma (HCC) accounts for a majority of primary liver cancer cases and related deaths. The purpose of this study was to assess the diagnostic value of splicing factor 3b subunit 4 (SF3B4) as a novel non-invasive biomarker for HCC and determine the association between *SF3B4* expression and immune cell infiltration.

**Methods:**

An enzyme-linked immunosorbent assay (ELISA) was used to detect SF3B4 levels in plasma samples obtained from healthy controls (HCs) and patients with chronic hepatitis, liver cirrhosis, and HCC. The expression levels of autoantibodies that detect SF3B4 in the plasma samples of each group of patients were measured. Small extracellular vesicles (EVs) were isolated from patient sera, and the expression levels of EV-*SF3B4* were measured using quantitative reverse transcription PCR.

**Results:**

ELISA results confirmed that the expression levels of SF3B4 proteins and autoantibodies in the plasma of patients with HCC were higher than those in HCs. However, their diagnostic performance was not better than that of alpha-fetoprotein (AFP). The mRNA expression of *SF3B4* in serum EV increased but not in the buffy coat or serum of patients with HCC. Serum EV-*SF3B4* displayed better diagnostic power than AFP for all stages of HCC (AUC = 0.968 vs. 0.816), including early-stage HCC (AUC = 0.960 vs. 0.842), and this was consistent in the external cohort. Single-cell RNA sequencing indicated that *SF3B4* expression was correlated with myeloid-derived suppressor cells. The Tumor Immune Estimation Resource database reconfirmed the correlation between *SF3B4* expression and immune cell infiltration in HCC.

**Conclusions:**

SF3B4 may be associated with tumor immune infiltration in HCC, and EV-*SF3B4* shows potential as a novel non-invasive diagnostic biomarker of HCC.

**Supplementary Information:**

The online version contains supplementary material available at 10.1186/s13046-023-02867-y.

## Background

Liver cancer was reportedly the sixth most commonly diagnosed cancer and the third leading cause of cancer-related deaths worldwide in 2020, with approximately 906,000 new cases and 830,000 deaths [1]. Hepatocellular carcinoma (HCC) is the most common histological type, accounting for a majority of cases and deaths owing to primary liver cancer. The main risk factors are chronic hepatitis B virus (HBV) or hepatitis C virus (HCV) infection, excessive alcohol consumption, non-alcoholic fatty liver disease, aflatoxin B1, and smoking [2].

Although various studies have deepened our understanding of HCC carcinogenesis, HCC surveillance mainly relies on abdominal ultrasonography and blood alpha-fetoprotein (AFP) measurements in high-risk groups with hepatitis B, hepatitis C, and liver cirrhosis (LC) [3–5]. It may be considered a type of carcinoma for which liquid biopsy is most urgently needed given the low sensitivity of abdominal ultrasonography for early HCC detection and the difficulties associated with liver biopsies in medical situations owing to high bleeding risks [6]. Several new blood-based biomarkers, including des-gamma-carboxy prothrombin, lens culinaris agglutinin-reactive AFP, plasma microRNA expression, methylated DNA markers, circulating tumor DNA, and circulating tumor cells, have been studied [7–12]. However, none of these markers have been included in standard surveillance methods in practice guidelines.

Splicing factor 3b subunit 4 (SF3B4) may function as a driver of HCC development and shows potential as a histological marker of HCC. SF3B4 regulates cell cycle expression and epithelial-mesenchymal transition proteins via spliceosome activity on the tumor suppressor *Kruppel-like factor 4* in HCC [13]. However, few studies have investigated the potential of SF3B4 as a marker in liquid biopsies pertaining to HCC [14].

This study evaluated the potential of SF3B4 as a liquid biopsy marker for HCC using several methods, such as protein, autoantibody, and RNA measurements. Overall, our results indicated that measurement of serum extracellular vesicle (EV)-derived *SF3B4* RNA delivered the best performance in regard to early HCC diagnostic ability and high positive rates in HCC, particularly in the absence of high AFP (> 20 ng/mL). In addition, SF3B4 was highly expressed in myeloid-derived suppressor cells (MDSCs). SF3B4 was highly positively correlated with five MDSC markers (ITGAM, LOX, S100A9, CD80, and CD83), wherein higher alterations of SF3B4 and the five markers correspond with poorer differentiation of HCC and worse prognosis of patients with HCC.

## Methods

### Expression and prognosis profiling of *SF3B4* in various cancer types

The Gene Expression Profiling Interactive Analysis (GEPIA) database (http://gepia.cancer-pku.cn/) containing gene expression data from The Cancer Genome Atlas liver hepatocellular carcinoma (TCGA_LIHC) and Genotype-Tissue Expression (GTEx) projects was used to profile *SF3B4* expression in various types of cancer. The differential expression of in various tumor samples was compared with that in adjacent non-tumor samples. We conducted overall survival (OS), disease-free survival (DFS), disease-specific survival (DSS), and progression-free survival (PFS) analyses to identify the prognostic performance of *SF3B4* expression levels in TCGA_LIHC. OS was defined as the time from HCC diagnosis to death from any cause; DFS was defined as the time from curative treatment to disease recurrence; DSS was defined as the time from HCC diagnosis to death owing to HCC alone; PFS was defined as the time from curative treatment to disease progression, relapse, or death due to any cause. The Human Protein Atlas (www.proteinatlas.org) was used to identify the positive staining rate of SF3B4 in HCC tissues.

### Patient enrollment and clinical term definitions

The blood samples and data of cohorts 1, 2, and 3 were provided by the Biobank of Ajou University Hospital, Suwon, South Korea, between January 2014 and December 2018. The study participants were divided into four groups. Healthy controls (HCs) were defined as patients aged 18–50 years who visited the Ajou Health Promotion Center for regular health checkups without any medical history. Chronic hepatitis (CH) was defined as the persistence of serum HBsAg or hepatitis C RNA levels for over six months. LC was diagnosed based on morphological changes observed via ultrasonography, liver stiffness detected via elastography, and measurements of platelet and albumin levels in the blood. HCC was diagnosed in accordance with the guidelines of the American Association for the Study of Liver Diseases. In this study, early-stage HCC was defined as a single lesion below 2 cm in diameter, corresponding to modified Union for International Cancer Control (mUICC) stage I. Clinical data containing information pertaining to age, sex, aspartate aminotransferase level, alanine aminotransferase level, platelet count, serum AFP level, etiology of liver disease, serum albumin level, serum bilirubin level, and international normalized ratio were recorded. Additionally, patients with HCC were investigated for the presence of vascular invasion and tumor stage based on Barcelona Clinic Liver Cancer and mUICC staging systems. Cohort 1 consisted of 45, 45, and 122 patients with CH, LC, and HCC, respectively, and 36 HCs; Cohort 2 comprised 25, 25, and 100 patients with CH, LC, and HCC, respectively, and 30 HCs. Cohort 3 comprised 26, 32, and 75 patients with CH, LC, and HCC, respectively, and 26 HCs. We collected additional samples from three different institutions for external validation: 30 patients with CH, 23 with LC, 59 with HCC, and 30 HCs. There were 29, 20, and 10 HCC samples corresponding to mUICC stages I, II, and III, respectively. These samples were sourced from the Bank of KIRAMS Radiation (Seoul, South Korea), Seoul National University Hospital Biobank (Seoul, South Korea), and Keimyung University Dongsan Hospital Biobank (Daegu, South Korea). The baseline characteristics of patients in each cohort are shown (Tables S1-S4). In addition, the combination analysis for AFP and EV-*SF3B4* was conducted by aggregating the Ajou University Hospital cohort and the external cohort (referred to as a multi-center cohort). Excluding patients with missing AFP values, the analysis was conducted on HC: 56 individuals, CH: 50 individuals, LC: 54 individuals, mUICC I: 61 individuals, mUICC II: 28 individuals, and mUICC III/IV: 43 individuals. All experiments performed in this study were conducted in accordance with the ethical guidelines of the 1975 Declaration of Helsinki. The study protocol was approved by the Institutional Review Board of the Ajou University Hospital, Suwon, South Korea (AJRIB-BMR-KSP-18-397, AJIRB-BMR-KSP-18-299, AJOUIRB-EX-2022-389, and AJOUIRB-EX-2023-368). The requirement for informed consent was waived.

### Prediction of SF3B4 and AFP derived in EV

The Exocarta (http://www.exocarta.org/) and exoRBase 2.0 (http://www.exorbase.org/) databases were used to identify the expression levels of *SF3B4* and *AFP* in human blood-derived small EVs in different diseases.

### Cell culture

The human HCC cell line, SNU449, was acquired from the Korean Cell Line Bank (Seoul, South Korea). Immortalized normal hepatocytes (MIHA) were provided by Dr. Roy-Chowdhury (Albert Einstein College of Medicine, Bronx, NY, USA). MIHA cells were cultured in Dulbecco’s modified Eagle’s medium (GenDEPOT, Barker, TX, USA), while SNU449 cells were cultured in RPMI1640 (GenDEPOT). Both mediums were supplemented with 10% fetal bovine serum (FBS) (Invitrogen, Waltham, MA, USA) and 100 U·mL^−1^ penicillin-streptomycin (GenDEPOT). Cells were grown in a humidified incubator at 37 °C and 5% CO_2_.

### Blood specimen preparation

Five milliliters of whole blood were collected from each individual directly into EDTA-containing tubes (for plasma and buffy coat) or serum-separator tubes (for serum). The blood was centrifuged at 2000 ×g for 5 min at 4 °C, and the resultant plasma, buffy coat, or sera were aliquoted into 1.5 mL tubes and stored at -80 °C until use. The serum samples were centrifuged at 3000 ×g at 4 °C for 15 min to remove cell debris before small EV isolation.

### Characterization of serum small EVs

We employed Exo-Quick (EXOQ5A-1, System Biosciences, Mountain View, CA, USA) to extract small EVs from serum which was sourced from patients and SNU449 cells according to the manufacturer’s guidelines. Serum samples and SNU449 culture media were combined with ExoQuick and incubated overnight at 4 °C. Subsequently, the patient-derived serum/ExoQuick mixture was centrifuged at 15,000 ×g for 2 min at 4 °C, while the cell culture media/ExoQuick mixture underwent centrifugation at 1,500 ×g for 30 min at 4 °C. Following the removal of the supernatant, we resuspended the pellet in DPBS.

Nanoparticle Tracking Analysis (NTA) was used to measure the size and quantity of isolated small EVs. A NanoSight NS300 (Malvern Panalytical Ltd., Malvern, UK) equipped with a 405 nm laser with a frame rate of 30 frames/s was used to record particle movements. Recorded videos were evaluated using NTA software (version 3.0, Malvern Panalytical). Each sample was analyzed thrice, and the counts were merged.

Small EV and SUN449 cell lysates were dissolved using radioimmunoprecipitation (RIPA) buffer (Thermo Scientific, Waltham, MA, USA) containing a Halt Protease Inhibitor Cocktail (Thermo Scientific). A bicinchoninic acid assay (Thermo Scientific) was used to quantify the total protein concentration. Subsequently, 10 µg protein was separated on a 4–20% Mini-PROTEAN TGX™ gel (Bio-Rad Laboratories, Hercules, CA, USA) and transferred onto polyvinylidene difluoride membranes (Amersham; GE Healthcare, Munich, Germany). The membranes were blocked using 5% non-fat milk in Tris-Buffer Saline (TBS) and 0.1% Tween-20 and immunoblotted utilizing the following primary antibodies: mouse anti-CD63 (1:1000, ab134045; Abcam, Cambridge, MA, USA), mouse anti-CD81 (1:250, 10630D; Invitrogen, Carlsbad, CA, USA), rabbit anti-CD9 (1:2000, ab92726; Abcam), and mouse anti-BiP/GRP78 (1:1000, 610979; BD Biosciences, San Jose, CA, USA). The samples were then incubated with secondary HRP-conjugated anti-rabbit (BR170-6515; Bio-Rad Laboratories) or anti-mouse (BR170-6516; Bio-Rad Laboratories) antibodies. Clarity Western ECL Substrate and a ChemiDoc imaging system (both from Bio-Rad Laboratories) were used to detect chemiluminescent signals.

### RNA isolation

Total RNA from cell lines was isolated using QIAzol reagent (Qiagen, Hilden, Germany) according to the manufacturer’s instructions. Buffy coat and serum RNA were isolated using TRIzol-LS reagent (Invitrogen) according to the manufacturer’s instructions. The buffy coat and serum were lysed in TRIzol-LS, with the RNA phase separated using chloroform, precipitated with 100% isopropanol, washed with 75% EtOH, and eluted in RNase-free water. Serum-derived EV RNA was extracted using a SeraMir™ Small EV RNA Amplification Kit (System Biosciences) according to the manufacturer’s instructions. EV lysates extracted from serum using Exo-Quick were mixed with lysis buffer and 100% EtOH. The mixtures were vortexed, transferred to a spin column, centrifuged at 15,000 ×g for 1 min, washed twice with wash buffer, and centrifuged again for 2 min; the serum-derived EV RNA was eluted in the elution buffer. RNA concentration was measured using a NanoPhotometer® N60 (Implen Inc., Westlake Village, CA, USA).

### 22 K human protein microarray

Autoantibodies that were highly expressed in HCC were identified by performing protein microarray experiments using a HuProt human proteome microarray v3.0 (CDI Laboratories Inc., Mayaguez, PR, USA). The protein chip was equilibrated with microarray buffer containing 137 mM NaCl, 2.7 mM KCl, 4.3 mM Na_2_HPO_4_, 1.8 mM KH_2_PO_4_ (pH 7.4), and 0.05% Triton X-100 for 5 min at room temperature (RT) and sequentially incubated with blocking solution composed of 5% Ig-G-free bovine serum albumin (BSA) (Merck KgaA, Darmstadt, Germany) in microarray buffer for 1 h at RT. To screen HCC-specific autoantibodies, a blocked protein chip was washed thrice with microarray buffer for 10 min, incubated with 20 µg/mL of serum in reaction buffer (50 mM Tris-Cl pH 7.5, 2 mM dithiothreitol (DTT), and 2.5 mM MgCl_2_) for 8 h at 4 °C, and washed with microarray buffer for 10 min. The washed protein chip was incubated with Alexa Fluor goat anti-rabbit 647-conjugated secondary antibodies (1:5000) diluted in microarray buffer containing 1% blocking solution for 30 min at RT. The chip was subsequently washed thrice with microarray buffer, dried via centrifugation in a 50-mL conical tube (200 ×g for 2 min at RT), and scanned using an Axon GenePix 4000 B microarray scanner (Molecular Devices, San Jose, CA, USA). All spotted proteins were probed using glutathione S-transferase antibodies (1:5000) and Alexa Fluor goat anti-rabbit 546-conjugated secondary antibodies. The signal intensity of each spot was recorded as the ratio of the foreground to background signal and normalized to that of glutathione S-transferase. The mean signal intensity of all the proteins on the chip was calculated.

### Enzyme-linked immunosorbent assay

Plasma SF3B4 levels were measured using a commercially available enzyme-linked immunosorbent assay (ELISA) kit (MBS9319293; MyBioSource Inc., San Diego, CA, USA) according to the manufacturer’s instructions. Plasma samples were diluted to 1:8 using sample dilution solution in the kit.

### Autoantibody detection

Autoantibody detection was performed via an ELISA using a 96-well microplate coated overnight at 4 °C with SF3B4 recombinant antigens. The remaining binding sites on the microplate were blocked with 20% casein buffer in distilled water. Ten microliters of freshly thawed patient or normal serum samples (diluted 1/250 in 20% casein buffer in PBS) were added to appropriate wells and incubated for 1 h at RT. Each well was washed thrice with PBS with 0.2% Triton X-100 (PBST; Sigma-Aldrich, St.Louis, MO, USA). Diluted horseradish peroxidase (HRP)-conjugated mouse anti-human IgG (Jackson Immuno Research, West Grove, PA, USA) was added to the target wells, and goat anti-mouse IgG was added to the calibrator wells for 1 h at RT. The well was washed with 0.2% PBS-T, and 100 µL of 3,39,5,59-tetramethylbenzidine (TMB; Thermo Fisher, MA, USA) substrate solution was added to each well, followed by incubation for 5 min at RT, and the addition of an equal volume of stopping solution (1 M HCl). The optical density was measured at 450 nm.

### IHC

Tissue samples fixed in formalin from patients with HCC were embedded in paraffin and sliced into 5-µm sections. The sections were deparaffinized using xylene, hydrated via an alcohol gradient, and incubated with primary antibodies (HPA028578, Atlas antibodies, Stockholm, Sweden) overnight at 4 °C. After washing thrice, the sections were incubated with secondary antibodies for 1 h. Finally, the sections were incubated until the desired staining intensity developed.

### Immunofluorescence

MIHA cells were seeded in 24-well plates and treated with SNU449-derived small EVs, while the controls were left untreated. Four hours later, the cells were fixed in 4% paraformaldehyde (Biosesang, Yongin, South Korea) for 10 min at 22–25 °C and permeabilized in PBST for 10 min. The cells were washed in PBST; the plates were blocked with 2.5% BSA in PBST for 30 min and incubated at 4 °C overnight with the following primary antibodies: EEA1 (1:200, 3288; Cell Signaling Technology, Danvers, MA, USA) and CD81 (1:250, 10630D; Invitrogen). The plates were washed and incubated with AlexaFluor-555-conjugated secondary antibodies and AlexaFluor-488-conjugated secondary antibodies for 2 h, stained with 4′,6-diamidino-2-phenylindole (DAPI), and analyzed using an Olympus IX71 microscope (Tokyo, Japan).

### Quantitative reverse transcription PCR

The mRNA expression levels in cell lines, buffy coat, serum, serum-derived EVs, and tissues were measured using quantitative reverse transcription PCR (qRT-PCR). Cell line RNA, buffy coat RNA, or serum RNA was reverse transcribed into complementary DNA using the PrimeScript™ RT Master Mix (TaKaRa Bio, Otsu, Japan), whereas serum-derived EV RNA was reverse transcribed using the PrimeScript™ RT Master Mix or the miScript II RT kit (QIAGEN, Hilden, Germany). qRT-PCR was performed using AmfiSure qGreen Q-PCR Master Mix (GenDEPOT) and monitored in real-time using the CFX Connect Real-Time PCR Detection System (Bio-Rad Laboratories, CA, USA) or Applied Biosystems 7300 Real-Time PCR System (Applied Biosystems, Foster City, CA, USA). HMBS was used as the internal control. The primer sequences used for *SF3B4* amplification were 5′-AGTCAACACCCACATGCCAA-3′ (forward) and 5′-GGGTCCAGGTTCCCAATGAAA-3′ (reverse); *AFP* primer sequences were 5′-ACCCACTGGAGATGAACAGTC-3′ (forward) and 5′-GCTGGAGTGGGCTTTTTGTG − 3′ (reverse); *LOX* primer sequences were 5′-TCGCTACACAGGACATCATGC-3′ (forward) and 5′-CAATGGATAAATCAGTGCCTGGTG-3′ (reverse); *ITGAM* primer sequences were 5′-TCCCGGAAAACTCAGAGGTC-3′ (forward) and 5′-TGAGGCCGTGAAGTTGAGAT-3′ (reverse); *S100A9* primer sequences were 5′-ATCAACACCTTCCACCAATACTCTG-3′ (forward) and 5′-AGGTTAGCCTCGCCATCAGC-3′ (reverse); *CD80* primer sequences were 5′-AGTGGAGTCTTACCCTGAAATC-3′ (forward) and 5′-CACTTCCCAGGTGCAAAACA-3′ (reverse); *CD83* primer sequences were 5′-GCCCACTTGTCCCACTATCT-3′ (forward) and 5′-TCATTAGCCCATGCAACAGC-3′ (reverse); and *HMBS* primer sequences were 5′- GGAGGGCAGAAGGAAGAAAACAG-3′ (forward) and 5′-CACTGTCCGTCTGTATGCGAG-3′ (reverse). A relative standard curve method (${2}^{-\varDelta \varDelta {C}_{t}}$) was used to determine the relative expression. All experiments were performed in quadruplicate.

### Single-cell RNA-sequencing

Unique molecular identifier (UMI) counts and metadata of filtered cells (*n* = 50,023) from patients with HCC and intrahepatic cholangiocarcinoma were obtained from the National Center for Biotechnology Information (NCBI) Gene Expression Omnibus (GEO) under accession code GSE151530. The Seurat package in R (v4.0.1), a free software environment available at http://www.r-project.org/, was used to perform log normalization, feature selection using method = ‘vst,’ scaling of data, principal component analysis (PCA), cell clustering, non-linear dimensional reduction (including Uniform Manifold Approximation and Projection [UMAP] plotting), and heatmap generation. Briefly, the most variable genes (*n* = 3,000) across the filtered cells were selected using the FindVariableFeatures function to perform PCA on scaled data. Subsequently, the FindNeighbors and FindClusters functions were used to construct a shared nearest neighbor (SNN) graph (‘dims = 1:30’) and cluster the cells (‘resolution = 1’), respectively. The RunUMAP function was used with 50 PCA dimensions (‘dims = 1:50’) to visualize cells on a 2-D UMAP plot. Cells annotated as ‘MDSCs’ were extracted and used to generate independent Seurat objects. The same parameters were applied for the functions mentioned above except the RunUMAP function, which uses 30 PCA dimensions (‘dims = 1:30’). The enrichGO function in the R clusterProfiler package (v3.18.1) was used to perform Gene Ontology (GO) analyses of the top 100 enriched genes. Enrichment analysis of the ‘The Molecular Signatures Database (MSigDB) Hallmark 2020’ database was performed using the enrichr function in the R enrichR package (v3.0).

### Immune infiltration analysis

The Tumor Immune Estimation Resource (TIMER) website (https://cistrome.shinyapps.io/timer/) contains comprehensive resources that enable investigations into the correlation between gene expression and immune cell infiltration via data pertaining to over 30 types of cancers in TCGA. The association between SF3B4 and tumor purity and the four types of immune cells (CD8 + T cells, natural killer (NK) cells, regulatory T cells, and MDSC) were plotted using TIMER. In addition, the correlation between SF3B4 and biomarkers of MDSCs was explored using the ‘correlation’ module.

### Statistical analysis

Data are presented as means ± standard deviations (SDs). Statistical significance of differences between two groups was assessed using paired Student’s t-test or unpaired Welch’s t-test using GraphPad Prism version 10.0 (GraphPad Software, San Diego, CA, USA). One-way analysis of variance (ANOVA) with Tukey’s post-hoc analysis was used for multiple comparisons among three groups. Kaplan–Meier survival curves were constructed to assess the significance of the prognostic power between two patient groups. Significant differences between survival curves were determined using the log-rank test. Receiver operating characteristic (ROC) and Cox proportional hazard regression analyses were performed using IBM SPSS software (IBM SPSS Statistics for Windows, version 22.0, released 2013, IBM, USA). ROC curves were analyzed to evaluate the sensitivity, specificity, and respective area under the curve (AUC) with 95% confidence intervals (CIs) for plasma SF3B4 protein, plasma anti-SF3B4 autoantibodies, and serum-derived EV-*SF3B4*. Univariate and multivariate Cox regression analyses were conducted to evaluate the independent prognostic value of *SF3B4* using the survival package in R. The likelihood-ratio test was used to confirm the hazard proportional assumption of the Cox regression model. Multivariate Cox regression analysis was performed to adjust for interactions between variables. Variables with *P* < 0.05 in the univariate Cox regression were included in the multivariate Cox regression analysis. All experiments were replicated at least thrice. Statistical significance was set at *P* < 0.05.

## Results

### *SF3B4* is overexpressed in HCC and related to the clinical outcome of HCC

**Fig. 1:**
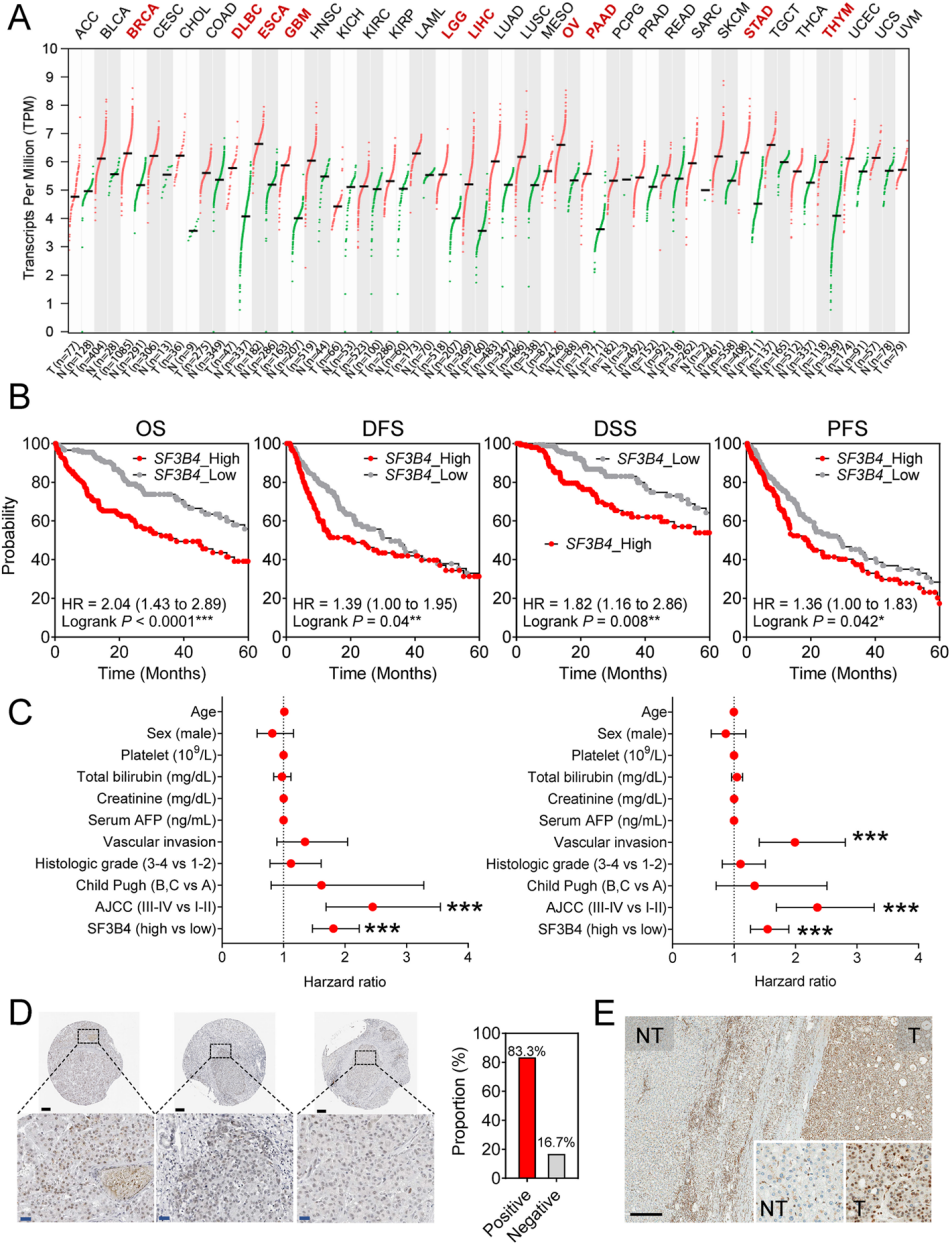
*SF3B4* is overexpressed in HCC and clinically correlated with its prognosis. **A ***SF3B4* expression in tumor and non-tumor tissues in the pan-cancer database of TCGA. **B** Kaplan–Meier survival analyses of *SF3B4* expression in TCGA_LIHC datasets for OS, DFS, DSS, and PFS. **C** Forest plot of univariate Cox regression analyses of clinical parameters for OS (left) and DFS (right) (***P <0.001). **D** Representative images of SF3B4 expression in HCC tissues and the proportion of patients with different SF3B4 immunostaining levels in liver cancer specimens based on HPA data. Scale bar = 100 µm. E. Representative IHC photomicrographs of SF3B4 in HCC (T, tumor tissues; NT, non-tumor tissues). Scale bar = 300 µm

Initially, we examined *SF3B4* expression in tumor and non-tumor tissues using the pan-cancer database, TCGA. Among the 33 different cancer types, *SF3B4* was significantly upregulated in tumor tissues compared with that in non-tumor tissues in 10 cancer types, including LIHC (Fig. 1A). Next, we assessed the association between *SF3B4* expression and prognosis among 10 different cancer types. Kaplan–Meier survival analysis indicated that only LIHC patients with high *SF3B4* expression had significantly lower OS and DFS than those with low *SF3B4* expression (Fig. S1A, B).

We performed survival analyses to further investigate the association between the prognoses of patients with HCC and the expression of *SF3B4*. Patients with high *SF3B4* expression had worse OS, DFS, DSS, and PFS (OS, log-rank *P* < 0.0001, hazard ratio [HR] = 2.04; DFS, log-rank *P* = 0.04, HR = 1.39; DSS, log-rank *P* = 0.008, HR = 1.82; PFS, log-rank *P* = 0.042, HR = 1.36; Fig. 1B). Univariate and multivariate Cox regression analyses demonstrated that high *SF3B4* expression was an independent poor prognostic factor for OS and DFS in patients with HCC (Fig. 1C, Table S5).

We explored the protein expression of SF3B4 in liver cancer tissues using the Human Protein Atlas database. Approximately 83% of the liver cancer tissues showed positive expression of SF3B4 (Fig. 1D). Similarly, we performed IHC analysis on non-tumor and tumor tissues from patients to confirm the expression of SF3B4. Consequently, SF3B4 was highly expressed in the nuclei of tumor tissues compared with that in non-tumor tissues (Fig. 1E).

### Expression levels of SF3B4 and anti-SF3B4 autoantibody as liquid biopsy biomarkers of HCC and their diagnostic performance in each cohort

**Fig. 2:**
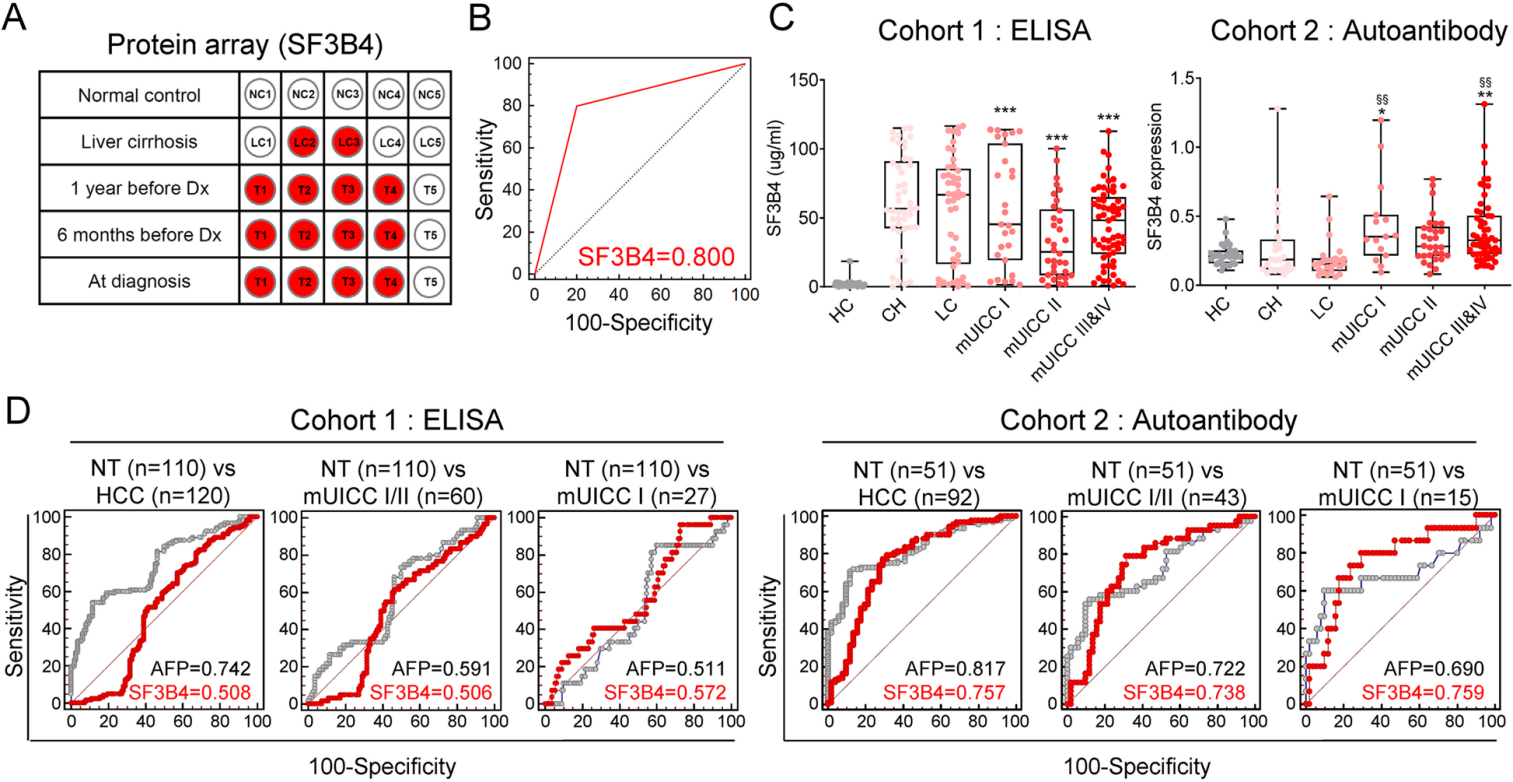
Expression of SF3B4 plasma protein and anti-SF3B4 autoantibodies as liquid biopsy biomarkers for HCC and their diagnostic performance in each cohort. **A** Positive dots of SF3B4 autoantibody expression from normal tissue and liver cirrhosis to HCC (Dx, HCC diagnosis). **B** ROC analysis of SF3B4 autoantibody. **C** Expression of plasma SF3B4 and expression of plasma anti-SF3B4 autoantibodies according to the stage of liver disease. Statistically significant differences were determined using one-way ANOVA with Tukey’s multiple comparisons test. Compared to HC; ***P <0.001, compared to LC; §§*P *<0.01. **D** AUCs of plasma SF3B4 in cohort 1 (left) and anti-SF3B4 autoantibody in cohort 2 (right) compared with serum AFP for diagnosing HCC. From left to right: diagnose all stages of HCC, mUICC stage I or II, and mUICC stage I

We analyzed 25 plasma samples from five healthy normal controls, five patients with LC, and five patients with early HCC (1 year before diagnosis, 6 months before diagnosis, and at diagnosis) using a 22 K protein chip to investigate the diagnostic potential of SF3B4 as a blood-based biomarker of HCC. All subjects were males with LC of HCC etiology involving HBV. Serial clustering analysis revealed that the SF3B4 autoantibody was positively expressed from LC to HCC groups but was undetected in the control group (Fig. 2A). Next, we performed ROC curve analysis to assess diagnostic performance. The SF3B4 autoantibody showed significant specificity and sensitivity as a biomarker, with an AUC of 0.8 (Fig. 2B).

Further validation involved measuring the expression levels of the SF3B4 protein and the anti-SF3B4 autoantibody in the plasma samples from two different cohorts. The baseline clinical characteristics of cohorts 1 and 2 are summarized (Tables S1, 2). Although SF3B4 protein expression in the plasma of patients with HCC was higher than that in HCs, there was no significant difference between patients with CH and those with LC or HCC. Similarly, anti-SF3B4 autoantibody expression was higher in the plasma samples from patients with HCC than in those from HCs (Fig. 2C). ROC curve analysis further showed that the diagnostic performance of plasma SF3B4 protein levels and anti-SF3B4 autoantibodies were lower than or similar to that of serum AFP (Fig. 2D).

### Serum-derived EV-*SF3B4* expression and its diagnostic power in HCC

**Fig. 3:**
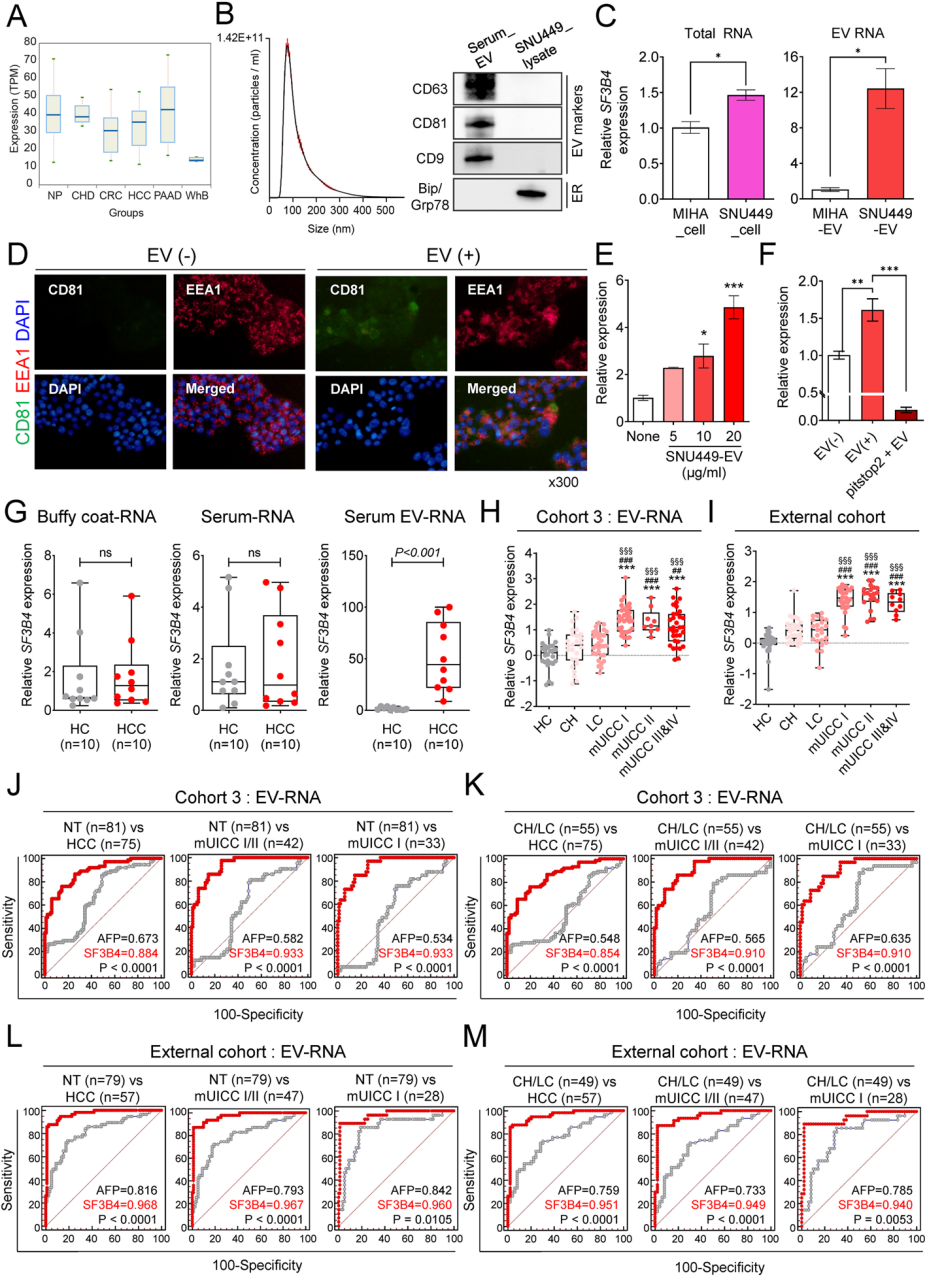
EV-*SF3B4* expression and its diagnostic power in HCC. **A** Box plot of *SF3B4* expression in blood-derived EVs from different diseases. NP, normal person; CHD, coronary heart disease; CRC, colorectal cancer; HCC, hepatocellular carcinoma; PAAD, pancreatic adenocarcinoma; WhB, whole blood; TPM, transcripts per million. **B** NTA of serum-derived EVs and Western blot analysis of EV and ER markers from serum EV and SNU449 cell lysates. **C** Gene expression of *SF3B4* in MIHA and SNU449 cell lysates and derived EVs. **D** Representative immunofluorescence of endocytic and EV markers from MIHA cells treated with SNU449-derived EVs. **E** The expression of *SF3B4* in MIHA cells treated with SNU449-derived EVs. **F** Effect of the inhibitor of clathrin-dependent endocytosis on decreasing *SF3B4* expression. **G*** SF3B4* mRNA expression comparison between healthy controls and patients with HCC in the buffy coat RNA (left), serum RNA (middle), and serum EV-RNA (right). **H** Expression of serum EV-*SF3B4* based on the stage of liver disease in cohort 3. Statistically significant differences were determined using one-way ANOVA with Tukey’s multiple comparisons test. Compared to HC; ***P <0.001, compared to CH; ##*P* <0.01, ###*P* <0.001, compared to LC; §§§*P* <0.001. **I** Expression of serum EV-*SF3B4* based on the stage of liver disease in the external cohort. Statistically significant differences were determined using one-way ANOVA with Tukey’s multiple comparisons test. Compared to HC; ***P <0.001, compared to CH; ###*P* <0.001, compared to LC; §§§*P* <0.001. **J** AUCs of serum EV-*SF3B4* compared to serum AFP for diagnosing HCC in cohort 3. From left to right: diagnose all stages of HCC, mUICC stage I or II, and mUICC stage I. **K** AUCs of serum EV-*SF3B4* for diagnosing HCC in the CH, LC, and HCC subgroups, compared to those of AFP in cohort 3. From left to right: diagnose all stages of HCC, mUICC stage I or II, and mUICC stage I. **L** AUCs of serum EV-*SF3B4* compared to serum AFP for diagnosing HCC in the external cohort. From left to right: diagnose all stages of HCC, mUICC stage I or II, and mUICC stage I. **M** AUCs of serum EV-*SF3B4* for diagnosing HCC in the CH, LC, and HCC subgroups compared to those of AFP in the external cohort. From left to right: diagnose all stages of HCC, mUICC stage I or II, and mUICC stage I

We used Exocarta and exoRBase 2.0 databases to confirm the expression of *SF3B4* in human blood-derived small EVs. Examination of these databases enabled us to predict that *SF3B4* would be expressed in blood-derived small EVs from HCC (Fig. 3A). Subsequently, EVs separated from the serum samples of patients with HCC were characterized using NTA and immunoblotting for positive and negative EV protein markers (Fig. 3B). Subsequently, qRT-PCR analysis revealed that *SF3B4* expression in the HCC cell line SNU449 was higher than that in the normal liver cell line, MIHA. Similarly, *SF3B4* expression in small EVs derived from the culture media was higher in SNU449 cells than in MIHA cells (Fig. 3C). Furthermore, immunofluorescence staining revealed that SNU449-derived small EVs were internalized by MIHA cells via endocytosis (Fig. 3D). *SF3B4* expression in MIHA cells increased with small EV concentration and decreased when the endocytosis inhibitor, pitstop2, was used (Fig. 3E, F).

We measured *SF3B4* expression in the buffy coat, serum, and serum-derived EVs from 10 HCs and 10 patients with HCC using qRT-PCR to confirm the expression of *SF3B4* in human blood components. *SF3B4* expression significantly increased in serum-derived EVs from patients with HCC (Fig. 3G). The expression level of serum EV-*SF3B4* in cohort 3 was examined to validate its diagnostic performance as an HCC predictor. Furthermore, we conducted validation analyses using samples from external institutions (external cohort) to mitigate the bias associated with a single institution study. Baseline characteristics of the cohorts are presented in Tables S3 and S4. Analysis of the expression of serum EV-*SF3B4* in cohort 3 and the external cohort according to the mUICC stage showed that serum EV-*SF3B4* expression gradually upregulated with tumor stage progression (Fig. 3H, I).

We compared the diagnostic performance of serum EV-*SF3B4* with that of AFP in cohort 3 to assess the diagnostic value of serum EV-*SF3B4* as a biomarker of HCC. The performance of serum EV-*SF3B4* in detecting HCC was excellent (AUC = 0.884) compared to that of AFP according to ROC curves (Fig. 3J). Additionally, serum EV-*SF3B4* levels had a high AUC (0.933) for early HCC (mUICC stage I). Moreover, serum EV-*SF3B4* exhibited a better AUC (0.854) than AFP (0.548) in the CH/LC vs. HCC model (Fig. 3K). Similarly, serum EV-*SF3B4* had a high AUC (0.910) for early HCC (mUICC stage I). Subsequently, we executed a rigorous validation of the diagnostic proficiency of EV-*SF3B4* against AFP utilizing ROC analysis in the external cohort. EV-*SF3B4* manifested superior AUC values in every comparison, despite AFP displaying elevated AUC values in relative comparisons within the external cohort. The AUC values were 0.816 for AFP and 0.968 for EV-*SF3B4* when compared against non-tumor (NT) conditions inclusive of HC, CH, and LC to HCC. The AUC values were 0.842 for AFP and 0.960 for EV-*SF3B4* between NT and mUICC I (Fig. 3L). Furthermore, the AUC values were identified as 0.759 for AFP and 0.951 for EV-*SF3B4* when excluding HC to concentrate on high-risk liver conditions, such as CH/LC, against HCC. The AUC values were 0.785 for AFP and 0.940 for EV-*SF3B4* when comparing CH/LC to mUICC I (Fig. 3M).

Subsequently, we explored the feasibility of EV-*AFP* as a diagnostic biomarker for HCC. We corroborated the expression of AFP employing the exoRBase 2.0 databases (Fig. S2A). Serum EV-*AFP* expression did not exhibit a progressive upregulation concomitant with tumor stage advancement, unlike EV-*SF3B4*. Serum EV-*AFP* manifested an inferior AUC (0.642) compared to the AUC of serum EV-*SF3B4* (0.968) (Fig. S2B).

### Diagnostic efficacy of the serum EV-*SF3B4* and serum AFP combination and the positive rate of serum EV-*SF3B4* at the early stages and all stages of HCC

**Fig. 4:**
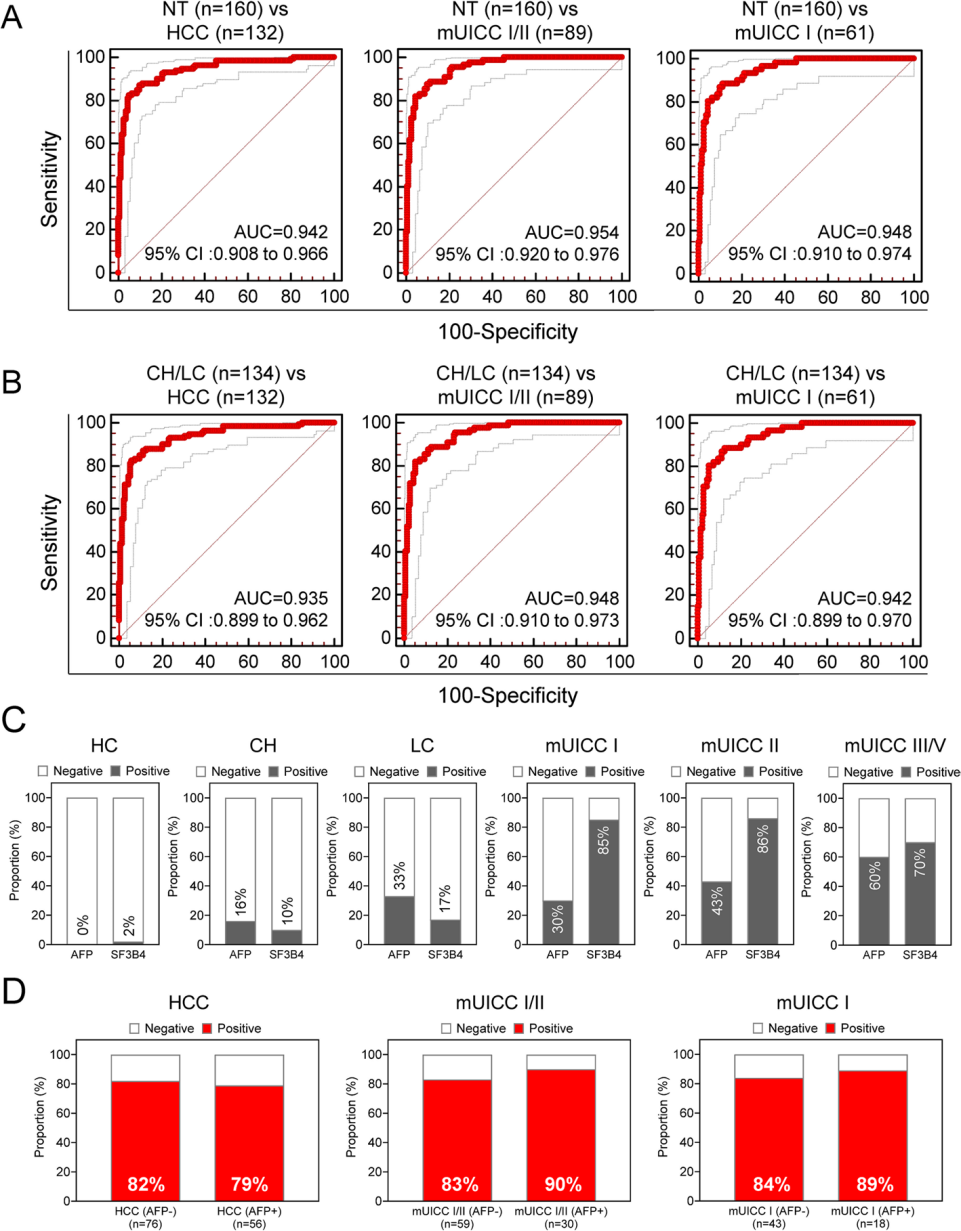
Efficiency of combined serum EV-*SF3B4* and AFP and EV-*SF3B4* positivity rates at all HCC stages in the multi-center cohort. **A** AUCs of the serum EV-*SF3B4* and AFP combination for diagnosing HCC in the multi-center cohort. From left to right: diagnoses of all stages of HCC, mUICC stage I or II, and mUICC stage I. **B** AUCs of the serum EV-*SF3B4* and serum AFP combination for diagnosing HCC in a subgroup composed of CH, LC, and HCC in the multi-center cohort. From left to right: diagnoses of all stages of HCC, mUICC stage I or II, and mUICC stage I. **C** Positive rates of AFP and serum EV-*SF3B4* in patients according to liver disease status in the multi-center cohort. **D** The rate of positive results for serum EV-*SF3B4* according to AFP status in patients with HCC, mUICC stage I or II, and mUICC stage I in the multi-center cohort

The combination analysis for AFP and EV-*SF3B4* was conducted by aggregating the Ajou University Hospital cohort and the external cohort (referred to as a multi-center cohort). The analysis was conducted on HC: 56 individuals, CH: 50 individuals, LC: 54 individuals, mUICC I: 61 individuals, mUICC II: 28 individuals, and mUICC III/IV: 43 individuals, excluding patients with missing AFP values. Analysis of the combination of AFP and EV-SF3B4 against NT and HCC, NT and mUICC I/II, and NT and mUICC I showed an AUC of 0.942, 0.954, and 0.948, respectively (Fig. 4A). Additionally, the AUC was 0.935, 0.948, and 0.942 when analyzed against CH/LC and HCC, CH/LC and mUICC I/II, and CH/LC and mUICC I, respectively (Fig. 4B).

Furthermore, examination of the positivity of AFP and EV-*SF3B4* in each disease group showed that AFP was 0% and EV-*SF3B4* was 2% in HC. In CH, AFP was 16% and EV-*SF3B4* was 10%. In LC, AFP was 33% and EV-*SF3B4* was 17%. In mUICC I, AFP was 30% and EV-*SF3B4* was 85%. In mUICC II, AFP was 43% and EV-*SF3B4* was 86%. In mUICC III/IV, AFP was 60% and EV-*SF3B4* was 70% (Fig. 4C). Finally, we confirmed high positivity of EV-*SF3B4* in patients with overall negative AFP in HCC at 82% (Fig. 4D, left), in mUICC I/II at 83% (Fig. 4D, middle), and in mUICC I at 84% (Fig. 4D, right).

### Correlation between *SF3B4* and immunity in the HCC microenvironment

**Fig. 5:**
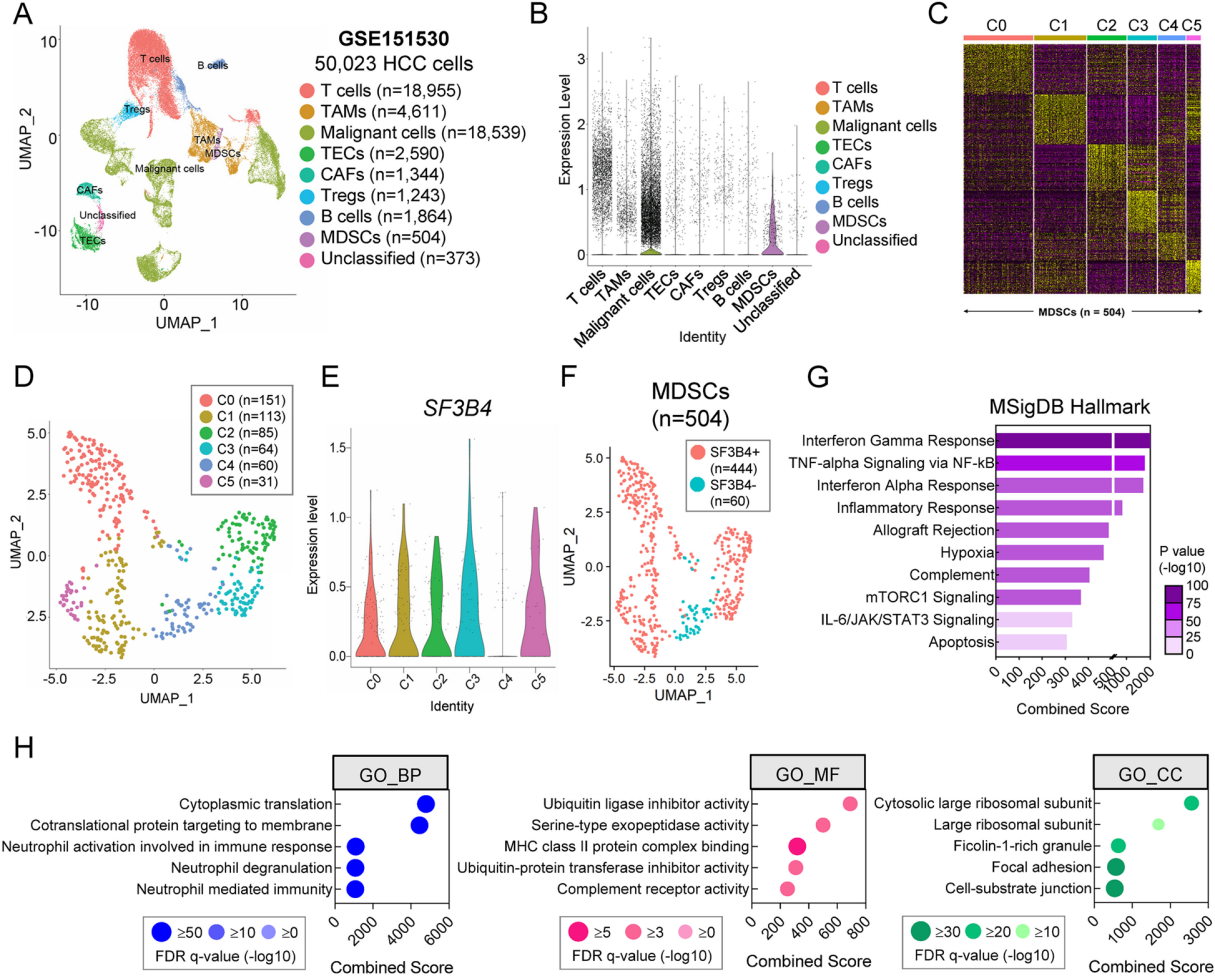
Single-cell RNA-seq analysis of *SF3B4* enrichment in the HCC microenvironment. **A** UMAP plots depict cells in the human HCC microenvironment. **B** Expression of *SF3B4* in each cell type. **C** Expression heatmap of the top 100 enriched genes in MDSC sub-clusters (C0–C5) (*n* = 504). **D** UMAP plot depicting MDSC subclusters. **E** Violin plot of the expression levels of *SF3B4 *in MDSC subclusters. **F** UMAP plot showing SF3B4+ and SF3B4- MDSCs. **G** Top 10 enriched terms in SF3B4 positive MDSC using the ‘MSigDB hallmark 2020’ database. **H** Gene Ontology depicts Biological Process (BP), Molecular Function (MF), and Cellular Component (CC) in SF3B4 positive MDSC

*SF3B4* was already investigated for its potential as a tissue marker that can be used to diagnose HCC, and the findings of this study further confirmed its potential as a non-invasive marker. However, the relationship between *SF3B4* expression and immunity in the HCC microenvironment has not been elucidated. We processed and analyzed a public scRNA-seq dataset (GSE151530) derived from patients with HCC to assess the differences between *SF3B4* expression levels across distinct immune cell populations. Nine cell types were identified among 50,023 QC-filtered HCC cells (Fig. 5A). Among these, *SF3B4* was highly expressed in MDSCs (Fig. 5B). The distribution of MDSCs was validated based on the distribution of five well-known MDSC markers (Fig. S3). MDSCs were further clustered into six subclusters, denoted as C0–C5 (Fig. 5C-E). *SF3B4* was overexpressed in all MDSC subclusters with the exception of C4. MDSCs were categorized into SF3B4 + and SF3B4- and visualized on a UMAP plot (Fig. 5F). The majority of the cells were SF3B4 positive. An analysis using the MSigDB was carried out on the genes enriched in SF3B4 positive cells, and the results are presented in Fig. 5G. Additionally, GO analysis was conducted on the SF3B4 positive cells to examine their biological processes, with the findings illustrated in Fig. 5H. Notably, the immune-related interferon gamma response pathway emerged as the most enriched term.

**Fig. 6:**
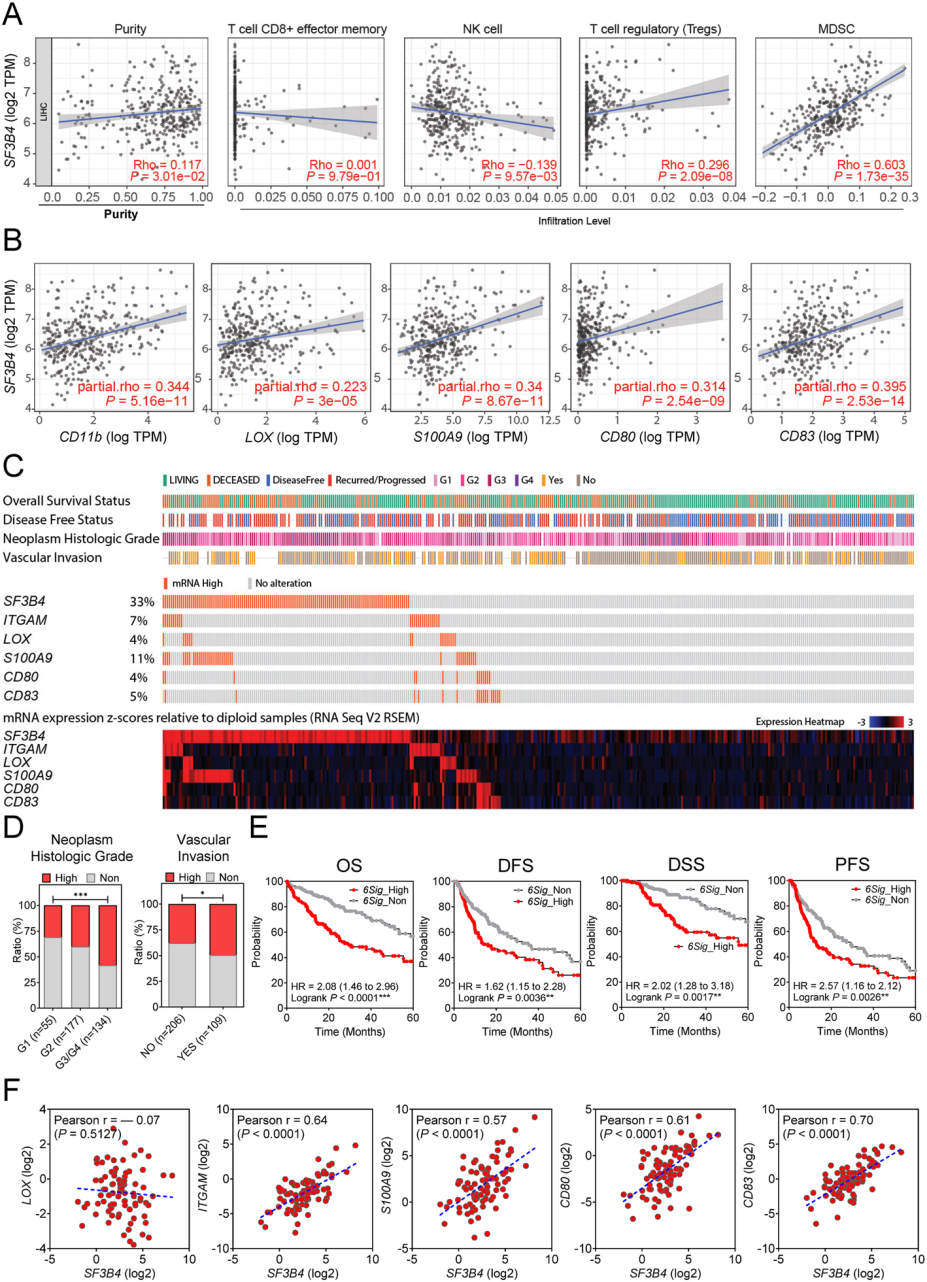
Correlation between *SF3B4* and MDSC markers and their clinical relevance in TCGA LIHC. **A** Correlation between *SF3B4 *expression and tumor-infiltrating immune cells. **B** Correlation between *SF3B4* expression and gene expression of MDSC markers. **C** OncoPrint of *SF3B4* and its highly correlated MDSC markers in patients with HCC using TCGA_LIHC dataset ordered by baseline characteristics (overall survival status, disease-free status, neoplasm histological grade, and vascular invasion), genetic alterations, and mRNA expression levels. **D** Bar charts showing the proportions of alteration levels of the six gene signatures in different clinical characteristics. **E** Kaplan–Meier survival analyses of the altered levels of six gene signatures (6 Sig) in TCGA_LIHC datasets for OS, DFS, DSS, and PFS. **F** Correlation between *SF3B4 *expression and MDSC markers in 86 human HCC tissues

The TIMER database was used to verify the correlation between *SF3B4* expression and tumor immune cell infiltration levels in HCC. Initially, we examined the relationship between different gene alterations in *SF3B4* and immune cell infiltration. CD8 + T cells, macrophages, neutrophils, and dendritic cell infiltration levels were associated with altered *SF3B4* gene copy numbers (Fig. S4). Furthermore, *SF3B4* expression was positively correlated with tumor purity, regulatory T cells, and MDSCs and negatively correlated with NK cells. *SF3B4* expression showed the strongest positive correlation with MDSCs according to scRNA-seq analysis (Fig. 6A). We investigated the markers of various types of immune cells (Table S6) to identify the correlation between SF3B4 and immune cell markers. The correlation between SF3B4 and each immune cell marker (Table 1) showed that *SF3B4* expression had a strong positive relationship with MDSCs. We further validated the association between *SF3B4* and MDSC marker expression. *ITGAM*, *LOX*, *S100A9*, *CD80*, and *CD83* MDSC markers showed significantly positive correlations with *SF3B4* (Fig. 6B). OncoPrints were illustrated for the clinical characteristics of patients and gene alteration levels of *SF3B4* and the five MDSC markers that showed a significant correlation with *SF3B4* based on the TCGA database (Fig. 6C). The proportion of patients with a high alteration level of the six-gene signatures increased as the histological grade increased. In addition, patients with vascular invasion showed a high rate of alterations in the six-gene signatures (Fig. 6D). Patients with high gene alteration levels experienced worse OS, DFS, DSS, and PFS (Fig. 6E). Finally, we validated the expression levels of *SF3B4* and MDSC markers in 86 pairs of HCC tissues using qRT-PCR. Bar graphs of the six genes are shown (Fig. S5). *SF3B4*, *LOX*, *ITGAM*, *S100A9*, *CD80*, and *CD83* overexpression was observed in 80 (93.0%), 42 (48.8%), 56 (65.1%), 32 (37.2%), 46 (53.5%), and 48 (55.8%) patients, respectively. The mRNA expression levels of *SF3B4* and other MDSC markers in HCC tissues of the same patient showed a high positive correlation (Pearson’s *r* = 0.64 for *ITGAM*, Pearson’s *r* = 0.57 for *S100A9*, Pearson’s *r* = 0.61 for *CD80*, and Pearson’s *r* = 0.70 for *CD83*), except for *LOX* (Fig. 6F).

**Table 1:** Immune cells related markers and correlation with *SF3B4*

Description	Markers	*SF3B4* (LIHC, 371)	
		**Corr**	***P* value**
**T cell (general)**	**CD3D**	**0.198**	**1.24e-04**
	**CD3E**	**0.11**	**3.99e-02**
	CD3G	0.096	6.38e-02
	**CD2**	**0.125**	**1.58e-02**
**Cytotoxic T cell**	CD8A	0.08	1.23e-01
	CD8B	0.088	8.97e-02
**Helper T cell**	CD4	-0.021	6.92e-01
**Follicular Helper T (Tfh) cells**	**CXCR5**	**0.158**	**2.33e-03**
	**ICOS**	**0.206**	**6.54e-05**
	**PD-1 (PDCD1)**	**0.256**	**5.7e-07**
	IL21	0.014	7.92e-01
	**CXCL13**	**0.119**	**2.14e-02**
	**BCL6**	**0.208**	**5.72e-05**
**T helper type 1 (Th1)**	**CCR5**	**0.136**	**8.71e-03**
	**CXCR3**	**0.185**	**3.41e-04**
	**IFG-gamma (IFNG)**	**0.173**	**7.97e-04**
	T-bet (TBX21)	-0.021	6.9e-01
	**STAT4**	**0.155**	**2.84e-03**
	**STAT1**	**0.266**	**2.1e-07**
**T helper type 2 (Th2)**	GATA3	0.091	8.03e-02
	**STAT6**	**0.195**	**1.6e-04**
	IL13	0.059	2.61e-01
**T helper type 17 (Th17)**	**STAT3**	**0.129**	**1.28e-02**
	IL17A	0.061	2.38e-01
**Regulatory T cell (Treg)**	**IL2RA**	**0.186**	**3.23e-04**
	**ENTPD1**	**0.271**	**1.26e-07**
	FoxP3	0.046	3.78e-01
	CCR8	0.231	6.89e-06
**T cell exhaustion**	**CTLA4**	**0.253**	**7.66e-07**
	**LAG3**	**0.177**	**6.07e-04**
	**TIM-3 (HAVCR2)**	**0.187**	**2.92e-04**
**Natural Killer (NK) cell**	**CD56 (NCAM1)**	**0.149**	**4.1e-03**
	**KLRD1**	**-0.116**	**2.51e-02**
	NKG2A (KLRC1)	0.039	4.59e-01
	NKG2D (KLRK1)	-0.026	6.12e-01
	NCR1 (NKp46)	-0.071	1.71e-01
	NCR2 (NKp44)	0.081	1.21e-01
	NCR3 (NKp30)	0.02	6.98e-01
**Pan Macrophage marker**	**CD68**	**0.127**	**1.42e-02**
**M1 macrophage**	**IL1B**	**0.273**	**9.62e-08**
	IL6	-0.025	6.36e-01
	**IL12A**	**0.355**	**1.9e-12**
	IL12B	0.047	3.72e-01
	**IL23A**	**0.14**	**6.73e-03**
	**TNF-alpha (TNF)**	**0.184**	**3.62e-04**
	INOS (NOS2)	0.032	5.43e-01
	**IRF5**	**0.462**	**5.67e-21**
	COX2 (PTGS2)	0.077	1.37e-01
**M2 macrophage**	CD163	-0.045	3.88e-01
	**PPARδ**	**0.448**	**1.11e-19**
	**PPARγ**	**0.262**	**2.98e-07**
	**MRC1 (CD206)**	**-0.167**	**1.25e-03**
	CD209	0.078	1.34e-01
	**IRF4**	**0.121**	**1.99e-02**
	**STAT3**	**0.129**	**1.27e-02**
	CCL17	0.1	5.43e-02
**Myeloid-Derived Suppressor Cells (MDSC)**	**HLA-DRA**	**0.125**	**1.64e-02**
	HLA-DRB1	0.083	1.09e-01
	**CD33**	**0.11**	**3.46e-02**
	**CD11b (ITGAM)**	**0.285**	**2.65e-08**
	**CD14 (negative)**	**-0.296**	**6.28e-09**
	IDO(IDO1)	0.068	1.89e-01
	**LOX**	**0.208**	**5.46e-05**
	S100A8	0.102	5.03e-02
	**S100A9**	**0.26**	**3.82e-07**
	**CD80**	**0.236**	**4.12e-06**
	**CD83**	**0.349**	**4.39e-12**
**B cell**	CD45 (PTPRC)	0.101	5.14e-02
	**CD19**	**0.217**	**2.56e-05**
	**CD79A**	**0.116**	**2.5e-02**
**Neutrophils**	CD66b (CEACAM8)	-0.024	6.51e-01
	CXCR1	0.054	3e-01
	**CXCR2**	**0.136**	**8.74e-03**
	MPO	-0.003	9.58e-01
**Dendritic cells**	IL3RA (CD123)	0.073	1.59e-01
	**ITGAX (CD11c)**	**0.204**	**7.81e-05**
	CD1c (BDCA1)	0.094	7.13e-02
	CLEC4C (BDCA2)	0.053	3.13e-01

## Discussion

SF3B4 is a subunit of the U2 small nuclear ribonucleoprotein that plays a crucial role in the splicing process, cell cycle regulation, cell differentiation, and immune deficiency-related activities [15]. It is associated with various carcinomas, such as pancreatic cancer, esophageal cancer, hematologic cancer, breast cancer, and ovarian cancer [16–20]. The oncogenic role played by SF3B4 in HCC was demonstrated via in vitro and in vivo experiments and the association between high expression levels of SF3B4 in HCC tissues and poor prognoses [13, 21]. miR-133 and serine and arginine-rich splicing factor 3 (SRSF3) may act as inhibitory regulators for SF3B4 [22, 23]. To date, only one study investigated the role of SF3B4 as a blood-based biomarker for diagnosing HCC. This study suggested that a twenty-gene-based gene set variation score (including peripheral blood mononuclear cell SF3B4) showed a good AUC (0.850) and enabled discrimination between patients with HCC and HC [14]. This study confirmed that serum EV-derived SF3B4 delivered the best HCC diagnostic performance after measuring SF3B4 levels in patients with HCC and control subjects using serum protein, plasma autoantibody, buffy coat RNA, serum RNA, and serum EV-RNA. Serum EV-*SF3B4* showed better performance in diagnosing early HCC (CH/LC vs. mUICC I; AUC, 0.910 and 0.940 for cohort 3 and external cohort, respectively) than AFP (AUC, 0.635 and 0.785 for cohort 3 and external cohort, respectively). EV-*SF3B4* showed a higher positivity rate (85%) than AFP (30%) in early HCC when positivity cut-off values of 7.81 fold were used for EV-*SF3B4* and 20 ng/mL for AFP. Moreover, EV-*SF3B4* demonstrated a high positivity rate (84%), even in AFP-negative early HCCs. The combination of AFP and serum EV-*SF3B4* exhibited an AUC of 0.942 (95% CI:0.908–0.966) in the HCC vs. non-tumor model and an AUC of 0.935 (95% CI:0.899–0.962) in the HCC vs. CH/LC model. These findings indicated that serum EV-*SF3B4* alone (or combined with AFP) shows clinical potential as a diagnostic biomarker for early HCCs.

Spliceosomes play a key role in cancer and immunity [24, 25]. Therefore, we investigated the relationship between SF3B4 and immune cells in the tumor microenvironment. Our analysis of public scRNA-seq datasets indicated that SF3B4 was highly expressed in MDSCs, which was confirmed via TIMER database analysis. Five MDSC markers (ITGAM, LOX, S100A9, CD80, and CD83) correlated well with SF3B4 expression, and these six gene signatures (SF3B4 and five MDSC markers) were significantly associated with HCC differentiation and patient prognoses in terms of OS, DFS, DSS, and PFS. Finally, we confirmed a 49–93% overexpression of SF3B4 and the five MDSC markers in 86 HCC tissues. To the best of our knowledge, this is the first study investigating the correlation between SF3B4 and immune cells in the tumor environment. In the tumor immune microenvironment, MDSCs exhibiting T-cell immunosuppressive functions differentiate into dendritic cells, tumor-associated macrophages, and granulocytes during tumor progression. MDSCs express pro-angiogenic factors, inducible carbon monoxide synthase, and indole 2, 3-dioxygenase. They play a role in promoting angiogenesis and in the inhibition of innate immunity and adaptive immunity [26, 27]. The role of SF3B4 in MDSCs remains unclear, although analysis of the MSigDB hallmark database suggests that the ‘interferon gamma response’ may be involved. SRSF3 acts as an inhibitory regulator of SF3B4 that affects the activity and function of dendritic cells via the SRSF3-pyruvate kinase M2 pathway, which is crucial for cancer cell migration and immune killing [28]. Thus, further studies may be needed to elucidate the entire role played by SF3B4 in MDSCs.

This study was affected by several limitations. Firstly, serum EV-*SF3B4* levels in patients with malignancies other than HCC were not measured. Consequently, we were unable to confirm the specificity of serum EV-*SF3B4* levels in HCC. Furthermore, slightly different cohorts (Cohort 1, 2, 3, and the external cohort) were used rather than the same cohort when implementing various measurement methods. This was unavoidable since only a limited number of samples were provided by the Biobank.

## Conclusions

The performance of serum EV-*SF3B4* levels regarding HCC diagnosis was excellent when used alone or combined with AFP. In particular, serum EV-*SF3B4* may play a diagnostic role in early HCC, wherein AFP is low (< 20 ng/ml). *SF3B4* is highly expressed in MDSCs in the tumor microenvironment, and the aberrant expression of *SF3B4* and MDSC markers may help predict poor prognosis in patients with HCC.

### Supplementary Information

**Additional file 1:** **Supplementary Table 1. **Clinicopathological characteristic of the patients in cohort 1. **Supplementary Table 2. **Clinicopathological characteristic of the patients in cohort 2. **Supplementary Table 3. **Clinicopathological characteristic of the patients in cohort 3. **Supplementary Table 4. **Clinicopathological characteristic of the patients in the external cohort. **Supplementary Table 5. **Univariate and multivariate Cox regression analyses of factors associated with overall survival and disease-free survival in TCGA LIHC dataset. **Supplementary Table 6. **Markers of immune cells. **Supplementary Fig. 1. **The correlation between the expression of *SF3B4* gene and the prognosis of ten different types of cancer in TCGA.A Overall survival rate and B Disease-free survival rate, as well as the expression of *SF3B*4 gene in various tumors using GEPIA2 software. **Supplementary Fig. 2. **EV-*AFP* expression and its diagnostic power in HCC. A Box plot of *AFP* expression in blood-derived EVs from different diseases. NP, normal person; CHD, coronary heart disease; CRC, colorectal cancer; HCC, hepatocellular carcinoma; PAAD, pancreatic adenocarcinoma; TPM, transcripts per million B Expression of serum EV-*AFP* based on the stage of liver disease (left) and AUCs of serum EV-*AFP* compared to serum EV-*SF3B4* for diagnosing HCC (right) in the external cohort. Statistically significant differences were determined using one-way ANOVA with Tukey’s multiple comparisons test. Compared to HC; *P <0.05, **P <0.01. **Supplementary Fig. 3. **The distribution of five well-known MDSC markers in whole cells. **Supplementary Fig. 4. **The relationship between *SF3B4* Copy Number Variation and immune infiltration. *P <0.05, **P<0.01. **Supplementary Fig. 5. **Bar chart showing the expression of *SF3B4* and MDSC markers in a set of 86 pairs of HCC tissues.

## Abbreviations

AFP: Alpha-fetoprotein
ANOVA: One-way analysis of variance
AUC: Area under the curve
CH: Chronic hepatitis
CI: Confidence interval
DAPI: 4′,6-diamidino-2-phenylindole
DFS: Disease-free survival
DSS: Disease-specific survival
ELISA: Enzyme-linked immunosorbent assay
GEO: Gene Expression Omnibus
GEPIA: Gene Expression Profiling Interactive Analysis
GO: Gene Ontology
GTEx: Genotype-Tissue Expression
HBV: Hepatitis B virus
HCC: Hepatocellular carcinoma
HCV: Hepatitis C virus
HRP: Horseradish peroxidase
LC: Liver cirrhosis
MDSCs: Myeloid-derived suppressor cells
MSigDB: Molecular Signatures Database
mUICC: Modified Union for International Cancer Control
NCBI: National Center for Biotechnology Information
NTA: Nanoparticle Tracking Analysis
OS: Overall survival
PCA: Principal component analysis
PFS: Progression-free survival
ROC: Receiver operating characteristic
SD: Standard deviation
SF3B4: Splicing factor 3b subunit 4
SNN: Shared nearest neighbor
TCGA: The Cancer Genome Atlas
TCGA_LIHC: The Cancer Genome Atlas liver hepatocellular carcinoma
TIMER: Tumor Immune Estimation Resource
TMB: 3,3',5,5'-Tetramethylbenzidine
UMAP: Uniform Manifold Approximation and Projection
UMI: Unique molecular identifier

## References

[1] Sung H, Ferlay J, Siegel RL, Laversanne M, Soerjomataram I, Jemal A (2021). Global Cancer statistics 2020: GLOBOCAN estimates of incidence and mortality worldwide for 36 cancers in 185 countries. CA Cancer J Clin.

[2] Zhang CH, Cheng Y, Zhang S, Fan J, Gao Q (2022). Changing epidemiology of hepatocellular carcinoma in Asia. Liver Int.

[3] European Association for the Study of the Liver (2018). Electronic address eee, European association for the study of the L. EASL clinical practice guidelines: management of hepatocellular carcinoma. J Hepatol.

[4] Marrero JA, Kulik LM, Sirlin CB, Zhu AX, Finn RS, Abecassis MM (2018). Diagnosis, staging, and management of hepatocellular carcinoma: 2018 practice guidance by the American association for the study of liver diseases. Hepatology.

[5] Vogel A, Meyer T, Sapisochin G, Salem R, Saborowski A (2022). Hepatocellular carcinoma. Lancet.

[6] Singal A, Volk ML, Waljee A, Salgia R, Higgins P, Rogers MA (2009). Meta-analysis: surveillance with ultrasound for early-stage hepatocellular carcinoma in patients with Cirrhosis. Aliment Pharmacol Ther.

[7] Marrero JA, Su GL, Wei W, Emick D, Conjeevaram HS, Fontana RJ (2003). Des-gamma carboxyprothrombin can differentiate hepatocellular carcinoma from nonmalignant chronic liver disease in American patients. Hepatology.

[8] Ahn JC, Teng PC, Chen PJ, Posadas E, Tseng HR, Lu SC (2021). Detection of circulating tumor cells and their implications as a biomarker for diagnosis, prognostication, and therapeutic monitoring in hepatocellular carcinoma. Hepatology.

[9] Borel F, Konstantinova P, Jansen PL (2012). Diagnostic and therapeutic potential of miRNA signatures in patients with hepatocellular carcinoma. J Hepatol.

[10] Kisiel JB, Dukek BA, R VSRK, Ghoz HM, Yab TC, Berger CK, et al. Hepatocellular carcinoma detection by plasma methylated DNA: discovery, phase I pilot, and phase II clinical validation. Hepatology. 2019;69:1180-92. 10.1002/hep.30244 PMC6429916 30168613

[11] von Felden J, Craig AJ, Villanueva A (2018). Role of circulating Tumor DNA to help decision-making in hepatocellular carcinoma. Oncoscience.

[12] Leerapun A, Suravarapu SV, Bida JP, Clark RJ, Sanders EL, Mettler TA (2007). The utility of Lens culinaris agglutinin-reactive alpha-fetoprotein in the diagnosis of hepatocellular carcinoma: evaluation in a United States referral population. Clin Gastroenterol Hepatol.

[13] Shen Q, Eun JW, Lee K, Kim HS, Yang HD, Kim SY (2018). Barrier to autointegration factor 1, procollagen-lysine, 2-oxoglutarate 5-dioxygenase 3, and splicing factor 3b subunit 4 as early-stage cancer decision markers and drivers of hepatocellular carcinoma. Hepatology.

[14] Lin Y, Liang R, Ye J, Li Q, Liu Z, Gao X (2019). A twenty gene-based gene set variation score reflects the pathological progression from Cirrhosis to hepatocellular carcinoma. Aging.

[15] Yan L, Yang X, Yang X, Yuan X, Wei L, Si Y (2021). The role of splicing factor SF3B4 in congenital diseases and tumors. Discov Med.

[16] Zhou W, Ma N, Jiang H, Rong Y, Deng Y, Feng Y (2017). SF3B4 is decreased in pancreatic cancer and inhibits the growth and migration of cancer cells. Tumour Biol.

[17] Kidogami S, Iguchi T, Sato K, Yoshikawa Y, Hu Q, Nambara S (2020). SF3B4 plays an oncogenic role in esophageal squamous cell carcinoma. Anticancer Res.

[18] Diao Y, Li Y, Wang Z, Wang S, Li P, Kong B (2022). SF3B4 promotes Ovarian cancer progression by regulating alternative splicing of RAD52. Cell Death Dis.

[19] La Starza R, Crescenzi B, Pierini V, Romoli S, Gorello P, Brandimarte L (2007). A common 93-kb duplicated DNA sequence at 1q21.2 in acute lymphoblastic leukemia and burkitt lymphoma. Cancer Genet Cytogenet.

[20] Denu RA, Burkard ME (2017). synchronous bilateral breast cancer in a patient with nager syndrome. Clin Breast Cancer.

[21] Iguchi T, Komatsu H, Masuda T, Nambara S, Kidogami S, Ogawa Y (2016). Increased copy number of the gene encoding SF3B4 indicates poor prognosis in hepatocellular carcinoma. Anticancer Res.

[22] Liu Z, Li W, Pang Y, Zhou Z, Liu S, Cheng K (2018). SF3B4 is regulated by microRNA-133b and promotes cell proliferation and metastasis in hepatocellular carcinoma. EBioMedicine.

[23] Lee J, Seo G, Hur W, Yoon SK, Nam SW, Lee JH (2020). SRSF3 depletion leads to an increase in SF3B4 expression in SNU-368 HCC cells. Anticancer Res.

[24] Yang H, Beutler B, Zhang D (2022). Emerging roles of spliceosome in cancer and immunity. Protein Cell.

[25] Foronda M (2021). RNA splicing meets anti-tumor immunity. Nat Cancer.

[26] Janols H, Bergenfelz C, Allaoui R, Larsson AM, Ryden L, Bjornsson S (2014). A high frequency of MDSCs in sepsis patients, with the granulocytic subtype dominating in gram-positive cases. J Leukoc Biol.

[27] Yu J, Du W, Yan F, Wang Y, Li H, Cao S (2013). Myeloid-derived suppressor cells suppress antitumor immune responses through IDO expression and correlate with lymph node metastasis in patients with breast cancer. J Immunol.

[28] Jiang H, Zhang S, Song T, Guan X, Zhang R, Chen X. Trichostatin a protects dendritic cells against oxygen-glucose deprivation via the SRSF3/PKM2/Glycolytic pathway. Front Pharmacol. 2018;9:612. 10.3389/fphar.2018.00612 PMC6004525 29942258

