# Supplementary material for: Circulating small extracellular vesicle-derived splicing factor 3b subunit 4 as a non-invasive diagnostic biomarker of early hepatocellular carcinoma
Source: J Exp Clin Cancer Res. 2023 Oct 30;42:288. doi: 10.1186/s13046-023-02867-y (PMC10614366; doi:10.1186/s13046-023-02867-y)
Supplement: Supplementary file 1 — Additional file 1: Supplementary Table 1. Clinicopathological characteristic of the patients in cohort 1. Supplementary Table 2. Clinicopathological characteristic of the patients in cohort 2. Supplementary Table 3. Clinicopathological characteristic of the patients in cohort 3. Supplementary Table 4. Clinicopathological characteristic of the patients in the external cohort. Supplementary Table 5. Univariate and multivariate Cox regression analyses of factors associated with overall survival and disease-free survival in TCGA LIHC dataset. Supplementary Table 6. Markers of immune cells. Supplementary Fig. 1. The correlation between the expression of SF3B4 gene and the prognosis of ten different types of cancer in TCGA.A Overall survival rate and B Disease-free survival rate, as well as the expression of SF3B4 gene in various tumors using GEPIA2 software. Supplementary Fig. 2. EV-AFP expression and its diagnostic power in HCC. A Box plot of AFP expression in blood-derived EVs from different diseases. NP, normal person; CHD, coronary heart disease; CRC, colorectal cancer; HCC, hepatocellular carcinoma; PAAD, pancreatic adenocarcinoma; TPM, transcripts per million B Expression of serum EV-AFP based on the stage of liver disease (left) and AUCs of serum EV-AFP compared to serum EV-SF3B4 for diagnosing HCC (right) in the external cohort. Statistically significant differences were determined using one-way ANOVA with Tukey’s multiple comparisons test. Compared to HC; *P <0.05, **P <0.01. Supplementary Fig. 3. The distribution of five well-known MDSC markers in whole cells. Supplementary Fig. 4. The relationship between SF3B4 Copy Number Variation and immune infiltration. *P <0.05, **P<0.01. Supplementary Fig. 5. Bar chart showing the expression of SF3B4 and MDSC markers in a set of 86 pairs of HCC tissues. [file 13046_2023_2867_MOESM1_ESM.docx]

**Supplementary Table 1. Clinicopathological characteristic of the patients in cohort 1**

AST, Aspartate aminotransferase; ALT, Alanine aminotransferase; AFP, $\alpha$-fetoprotein; INR, International Normalized Ratio

| Variables | Total cohort (n = 248) | | | |
| --- | --- | --- | --- | --- |
|  | HC (n = 36) | CH (n = 45) | LC (n = 45) | HCC (n = 122) |
| Age (years), mean ± SD | 43.8 ± 14.8 | 44.9 ± 6.9 | 52.7 ± 9.9 | 55.3 ± 9.7 |
| Male sex, n (%) | 27 (75) | 39 (86.7) | 41 (91.1) | 110 (90.2) |
| AST, IU/ml | 19.0 ± 4.5 | 68.9 ± 66.8 | 81.3 ± 136.0 | 62.2 ± 65.1 |
| ALT, IU/ml | 14.2 ± 7.2 | 94.3 ± 130.0 | 64.7 ± 105.9 | 45.7 ± 36.7 |
| Platelet, x10^9^/L | 244.7 ± 35.9 | 178.3 ± 43.3 | 136.3 ± 56.2 | 157.0 ± 62.7 |
| AFP (ng/mL), mean ± SD | 1.8 ± 0.6 | 10.8 ± 16.6 | 6.9 ± 14.9 | 7551.1 ± 18759.3 |
| Albumin (g/dL), mean ± SD |  | 4.5 ± 0.3 | 4.4 ± 0.5 | 4.4 ± 0.5 |
| Bilirubin (mg/dL), mean ± SD |  | 0.7 ± 0.2 | 1.3 ± 1.6 | 1.6 ± 3.2 |
| INR, mean ± SD |  | 1.1 ± 0.1 | 1.2 ± 0.1 | 1.1 ± 0.1 |
| BCLC stage, n (%) |  |  |  |  |
| 0 |  |  |  | 1 (11.1) |
| A |  |  |  | 3 (33.3) |
| B |  |  |  | 0 (0) |
| C |  |  |  | 5 (55.6) |
| D |  |  |  | 0 (0) |
| Modified UICC stage, n (%) |  |  |  |  |
| I |  |  |  | 27 (26.5) |
| II |  |  |  | 31 (30.4) |
| III |  |  |  | 21 (20.6) |
| IVA |  |  |  | 19 (18.6) |
| IVB |  |  |  | 4 (3.9) |
| Vascular invasion, n (%) |  |  |  | 26 (38.2) |

**Supplementary Table 2. Clinicopathological characteristic of the patients in cohort 2**

| Variables | Total cohort (n = 180) | | | |
| --- | --- | --- | --- | --- |
|  | HC (n = 30) | CH (n = 25) | LC (n = 25) | HCC (n = 100) |
| Age (years), mean ± SD | 52.8 ± 12.1 | 44.6 ± 10.1 | 49.6 ± 5.9 | 54.7 ± 8.9 |
| Male sex, n (%) | 29 (96.7) | 25 (100) | 25 (100) | 100 (100) |
| AST, IU/ml | 39.3 ± 82.7 | 73.8 ± 152.4 | 97.0 ± 149.7 | 69.7 ± 89.9 |
| ALT, IU/ml | 30.5 ± 36.5 | 121.6 ± 293.4 | 88.0 ± 127.9 | 52.0 ± 65.7 |
| Platelet, x10^9^/L | 231.5 ± 19.5 | 200.9 ± 46.4 | 135.1 ± 49.9 | 160.5 ± 72.1 |
| AFP (ng/mL), mean ± SD | 2.6 ± 1.0 | 7.2 ± 10.9 | 17.5 ± 34.2 | 4929.3 ± 14754.7 |
| Albumin (g/dL), mean ± SD |  | 4.6 ± 0.3 | 4.2 ± 0.6 | 4.1 ± 0.6 |
| Bilirubin (mg/dL), mean ± SD |  | 1.2 ± 2.0 | 2.0 ± 3.6 | 1.3 ± 2.6 |
| INR, mean ± SD |  | 1.1 ± 0.1 | 1.2 ± 0.1 | 1.2 ± 0.2 |
| BCLC stage, n (%) |  |  |  |  |
| 0 |  |  |  | 2 (13.3) |
| A |  |  |  | 7 (46.7) |
| B |  |  |  | 0 (0) |
| C |  |  |  | 6 (40) |
| D |  |  |  | 0 (0) |
| Modified UICC stage, n (%) |  |  |  |  |
| I |  |  |  | 12 (12) |
| II |  |  |  | 34 (34) |
| III |  |  |  | 24 (24) |
| IVA |  |  |  | 22 (22) |
| IVB |  |  |  | 8 (8) |
| Vascular invasion, n (%) |  |  |  | 14 (40) |

AST, Aspartate aminotransferase; ALT, Alanine aminotransferase; AFP, $\alpha$-fetoprotein; INR, International Normalized Ratio

**Supplementary Table 3. Clinicopathological characteristic of the patients in cohort 3**

| Variables | Total cohort (n = 159) | | | |
| --- | --- | --- | --- | --- |
|  | HC (n = 26) | CH (n = 26) | LC (n = 32) | HCC (n = 75) |
| Age (years), mean ± SD | 34.1 ± 8.1 | 46.5 ± 10.8 | 52.8 ± 10.0 | 54.9 ± 8.9 |
| Male sex, n (%) | 3 (11.5) | 13 (50.0) | 17 (53.1) | 58 (77.3) |
| AST, IU/ml | 16 ± 3.4 | 56.8 ± 53.3 | 81.5 ± 97.7 | 71.0 ± 91.8 |
| ALT, IU/ml | 13.6 ± 5.6 | 70.2 ± 79.9 | 74.3 ± 92.6 | 50.0 ± 63.3 |
| Platelet, x10^9^/L | 330.4 ± 52.1 | 181.5 ± 46.4 | 129.5 ± 76.4 | 160.0 ± 75.3 |
| AFP (ng/mL), mean ± SD | 1.8 ± 0.7 | 20.6 ± 25.4 | 62.2 ± 128.3 | 3477.9 ± 12752.0 |
| Etiology, n (%) |  |  |  |  |
| HBV |  |  |  | 67 (89.3) |
| HCV |  |  |  | 4 (5.3) |
| Alcohol |  |  |  | 3 (4.0) |
| Others |  |  |  | 1 (1.3) |
| Albumin (g/dL), mean ± SD |  | 4.5 ± 0.4 | 4.0 ± 0.5 | 4.3 ± 0.5 |
| Bilirubin (mg/dL), mean ± SD |  | 0.8 ± 0.3 | 1.1 ± 1.0 | 1.6 ± 4.1 |
| INR, mean ± SD |  | 1.2 ± 0.2 | 1.2 ± 0.2 | 1.2 ± 0.2 |
| BCLC stage, n (%) |  |  |  |  |
| 0 |  |  |  | 24 (32.0) |
| A |  |  |  | 18 (24.0) |
| B |  |  |  | 3 (4.0) |
| C |  |  |  | 30 (40.0) |
| D |  |  |  | 0 (0) |
| Modified UICC stage, n (%) |  |  |  |  |
| I |  |  |  | 33 (44) |
| II |  |  |  | 9 (12) |
| III |  |  |  | 11 (14.7) |
| IVA |  |  |  | 16 (21.3) |
| IVB |  |  |  | 6 (8.0) |
| Vascular invasion, n (%) |  |  |  | 24 (42.4) |
| Recurrence, n (%) |  |  |  | 31 (41.3) |
| OS (months), mean ± SD |  |  |  | 40.4 ± 19.8 |
| RFS (months), mean ± SD |  |  |  | 31.8 ± 22.0 |

AST, Aspartate aminotransferase; ALT, Alanine aminotransferase; AFP, $\alpha$-fetoprotein; HBV, Hepatitis B virus; HCV, Hepatitis C virus; INR, International Normalized Ratio; OS, Overall Survival; RFS, Recurrence Free Survival

**Supplementary Table 4. Clinicopathological characteristic of the patients in the external cohort**

| Variables | Total cohort (n = 142) | | | |
| --- | --- | --- | --- | --- |
|  | HC (n = 30) | CH (n = 30) | LC (n = 23) | HCC (n = 59) |
| Age (years), mean ± SD | 41.23 ± 13.54 | 39.97 ± 13.09 | 57.65 ± 9.54 | 57.34 ± 9.26 |
| Male sex, n (%) | 8 (26.67) | 17 (56.67) | 12 (52.17) | 45 (76.27) |
| AST, IU/ml | 19.53 ± 5.43 | 68.31 ± 88.09 | 47.04 ± 57.50 | 43.85 ± 20.69 |
| ALT, IU/ml | 15.67 ± 6.71 | 118.59 ± 269.34 | 46.00 ± 73.76 | 41.14 ± 27.56 |
| Platelet, x10^9^/L | 242.03 ± 54.01 | 226.75 ± 106.91 | 134.43 ± 43.01 | 164.39 ± 66.87 |
| AFP (ng/mL), mean ± SD | 2.65 ± 0.87 | 35.57 ± 93.62 | 72.31 ± 311.74 | 6450.23 ± 27936.65 |
| Albumin (g/dL), mean ± SD | 4.61 ± 0.25 | 4.10 ±0.37 | 3.75 ± 0.78 | 4.09 ± 0.37 |
| Bilirubin (mg/dL), mean ± SD | 0.68 ± 0.23 | 0.96 ± 0.50 | 1.22 ± 0.69 | 0.73 ± 0.53 |
| INR, mean ± SD |  | 1.09 ± 0.26 | 1.18 ± 0.25 | 1.11 ± 0.10 |
| Modified UICC stage, n (%) |  |  |  |  |
| I |  |  |  | 29 (49.15) |
| II |  |  |  | 20 (33.90) |
| III |  |  |  | 10 (16.95) |
| IVA |  |  |  | 0 (0) |
| IVB |  |  |  | 0 (0) |
| Vascular invasion, n (%) |  |  |  | 19 (32.20) |

AST, Aspartate aminotransferase; ALT, Alanine aminotransferase; AFP, $\alpha$-fetoprotein; INR, International Normalized Ratio

**Supplementary Table 5. Univariate and multivariate Cox regression analyses of factors associated with overall survival and disease-free survival in TCGA LIHC dataset.**

|  | OS | | | | | | DFS | | | | | |
| --- | --- | --- | --- | --- | --- | --- | --- | --- | --- | --- | --- | --- |
|  | **Univariate** | | | **Multivariate** | | | **Univariate** | | | **Multivariate** | | |
| Factor | **HR** | **95% CI** | ***P value*** | **HR** | **95% CI** | ***P value*** | **HR** | **95% CI** | ***P value*** | **HR** | **95% CI** | ***P value*** |
| Age | 1.012 | 0.999-1.026 | 0.775 |  |  |  | 0.997 | 0.985-1.009 | 0.625 |  |  |  |
| Sex (male) | 0.816 | 0.573-1.163 | 0.260 |  |  |  | 0.866 | 0.632-1.187 | 0.372 |  |  |  |
| Serum AFP (ng/mL) | 1.000 | 1.000-1.000 | 0.432 |  |  |  | 1.000 | 1.000-1.000 | 0.283 |  |  |  |
| Platelet (10^9^/L) | 1.000 | 1.000-1.000 | 0.711 |  |  |  | 1.000 | 1.000-1.000 | 0.763 |  |  |  |
| Total bilirubin (mg/dL) | 0.973 | 0.845-1.122 | 0.708 |  |  |  | 1.046 | 0.959-1.141 | 0.308 |  |  |  |
| Creatinine (mg/dL) | 1.002 | 0.986-1.018 | 0.804 |  |  |  | 1.002 | 0.987-1.017 | 0.819 |  |  |  |
| Child Pugh  (B,C vs A) | 1.616 | 0.797-3.275 | 0.183 |  |  |  | 1.334 | 0.710-2.507 | 0.371 |  |  |  |
| Histologic grade  (3-4 vs 1-2) | 1.120 | 0.781-1.606 | 0.539 |  |  |  | 1.106 | 0.811-1.507 | 0.525 |  |  |  |
| AJCC  (III-IV vs I-II) | 2.449 | 1.689-3.549 | ***2.3E-06*** | 2.290 | 1.577-3.325 | ***1.34E-05*** | 2.355 | 1.692-3.276 | ***3.7E-07*** | 1.913 | 1.269-2.883 | ***0.002*** |
| Vascular  invasion | 1.348 | 0.890-2.042 | 0.159 |  |  |  | 1.990 | 1.407-2.814 | ***9.9E-05*** | 1.607 | 1.106-2.334 | ***0.013*** |
| *SF3B4*  expression | 1.810 | 1.466-2.234 | ***3.33E-08*** | 1.725 | 1.371-2.169 | ***3.18E-06*** | 1.546 | 1.265-1.889 | ***2.04E-05*** | 1.391 | 1.084-1.786 | ***0.010*** |

**Supplementary Table 6. Markers of immune cells**

| **Description** | | **markers** | **Reference** |
| --- | --- | --- | --- |
| **T cell (general)** | | CD3D | [1] |
|  |  | CD3E |  |
|  |  | CD3G |  |
|  |  | CD2 | [2] |
| **Cytotoxic T cell** | | CD8A | [3] |
|  |  | CD8B |  |
| **Helper T cell** | | CD4 |  |
|  | **Follicular Helper T (Tfh) cells** | CXCR5 | [4], [5], [6] |
|  |  | ICOS | [4], [5] |
|  |  | PD-1 (PDCD1) | [4] |
|  |  | IL21 | [4], [5], [7] |
|  |  | CXCL13 | [6] |
|  |  | BCL6 | [4], [5], |
|  | **T helper type 1 (Th1)** | CCR5 | [8] |
|  |  | CXCR3 | [8] |
|  |  | IFN-gamma (IFNG) | [5] |
|  |  | T-bet (TBX21) | [7], [9], [10] |
|  |  | STAT4 | [11] |
|  |  | STAT1 | [10] |
|  | **T helper type 2 (Th2)** | GATA3 | [7], [9], [11] |
|  |  | STAT6 | [11] |
|  |  | IL13 | [5] |
|  | **T helper type 17 (Th17)** | STAT3 | [12] |
|  |  | IL17A | [5], [12] |
| **Regulatory T cell (Treg)** | | IL2RA (CD25) | [13], [14] |
|  |  | ENTPD1 (CD39) | [14] |
|  |  | FoxP3 | [5], [7], [13] |
|  |  | CCR8 | [15] |
| **T cell exhaustion** | | CTLA4 | [16] |
|  |  | LAG3 |  |
|  |  | TIM-3 (HAVCR2) |  |
| **Natural Killer (NK) cell** | | CD56 (NCAM1) | [17] |
|  |  | KLRD1 (CD94) | [18] |
|  |  | NKG2A (KLRC1) | [18] |
|  |  | NKG2D (KLRK1) | [18] |
|  |  | NCR1 (NKp46) | [18], [19] |
|  |  | NCR2 (NKp44) | [18], [20] |
|  |  | NCR3 (NKp30) | [21] |
| **Pan Macrophage marker** | | CD68 | [22] |
| **M1 macrophage** | | IL1 | [23] |
|  |  | IL6 | [23], [24] |
|  |  | IL12A | [23], [25] |
|  |  | IL12B |  |
|  |  | IL23 | [23] |
|  |  | TNF-alpha (TNF) | [23], [24] |
|  |  | INOS (NOS2) | [23], [25], [24] |
|  |  | IRF5 | [25] |
|  |  | COX2 (PTGS2) | [24] |
| **M2 macrophage** | | CD163 | [26] |
|  |  | PPAR$\delta$ |  |
|  |  | PPAR$\gamma$ |  |
|  |  | MRC1 (CD206) | [24], [26] |
|  |  | CD209 | [26] |
|  |  | IRF4 |  |
|  |  | STAT3 |  |
|  |  | CCL17 |  |
|  |  | IL-10 | [23], [25], [26] |
| **Myeloid-Derived Suppressor Cells (MDSC)** | | HLA-DRA (negative) | [27] |
|  |  | HLA-DRB1 (negative) |  |
|  |  | CD33 |  |
|  |  | CD11b (ITGAM) |  |
|  |  | CD14 (negative) |  |
|  |  | IDO (IDO1) | [28] |
|  |  | LOX | [29] |
|  |  | S100A8 | [30] |
|  |  | S100A9 |  |
|  |  | CD80 | [31] |
|  |  | CD83 |  |
| **B cell** | | CD45 (PTPRC) | [32] |
|  |  | CD19 | [33] |
|  |  | CD79A | [34] |
| **Neutrophils** | | CD66b (CEACAM8) | [35] |
|  |  | CXCR1 | [36] |
|  |  | CXCR2 |  |
|  |  | MPO | [37] |
| **Dendritic cells** | | IL3RA (CD123) | [38] |
|  |  | ITGAX (CD11c) |  |
|  |  | BDCA-1 | [39] |
|  |  | BDCA-2 (CD1C) | [40] |


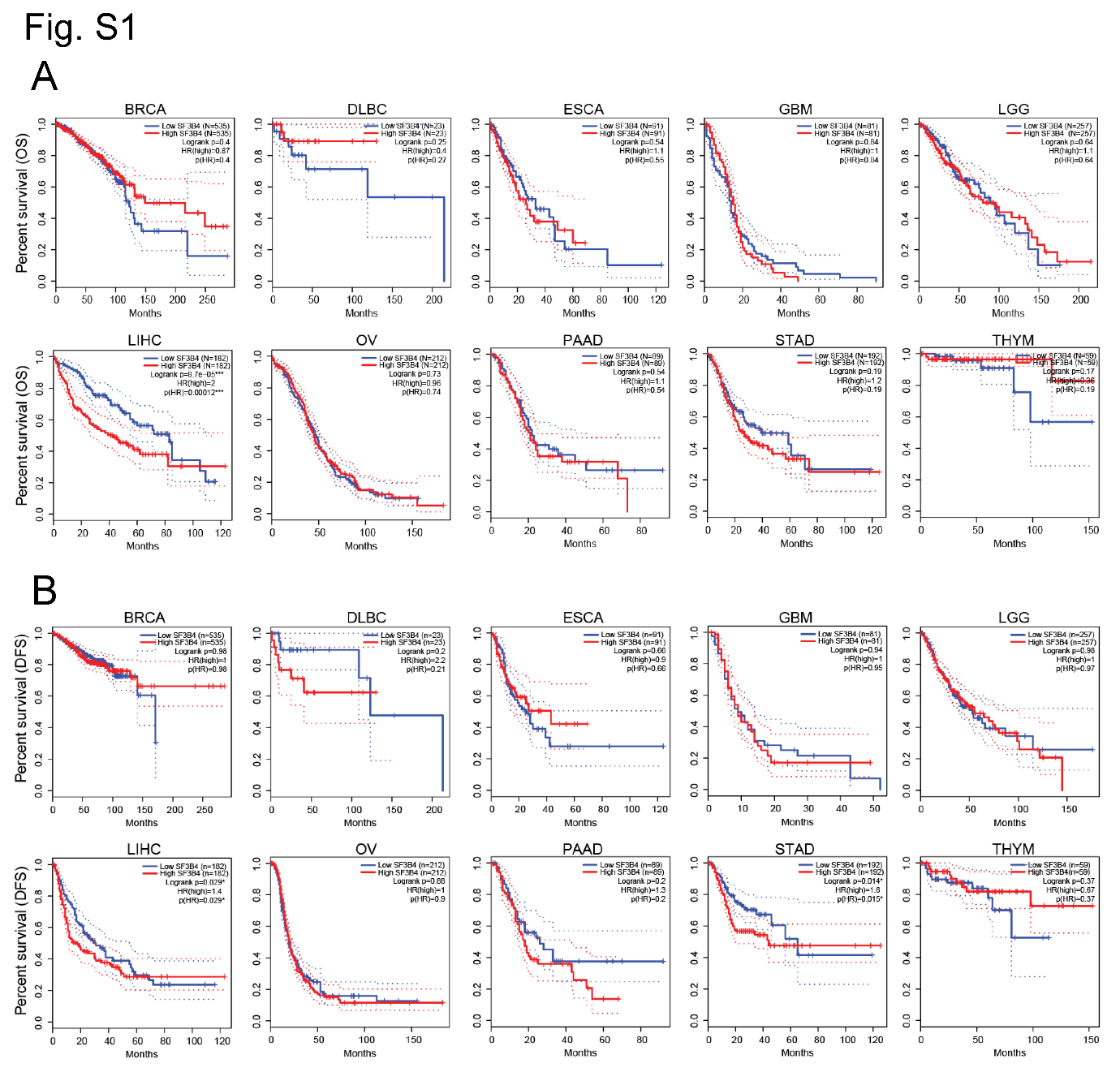


**Supplementary Fig. 1.** The correlation between the expression of *SF3B4* gene and the prognosis of ten different types of cancer in TCGA. **A** Overall survival rate and **B** Disease-free survival rate, as well as the expression of *SF3B*4 gene in various tumors using GEPIA2 software.


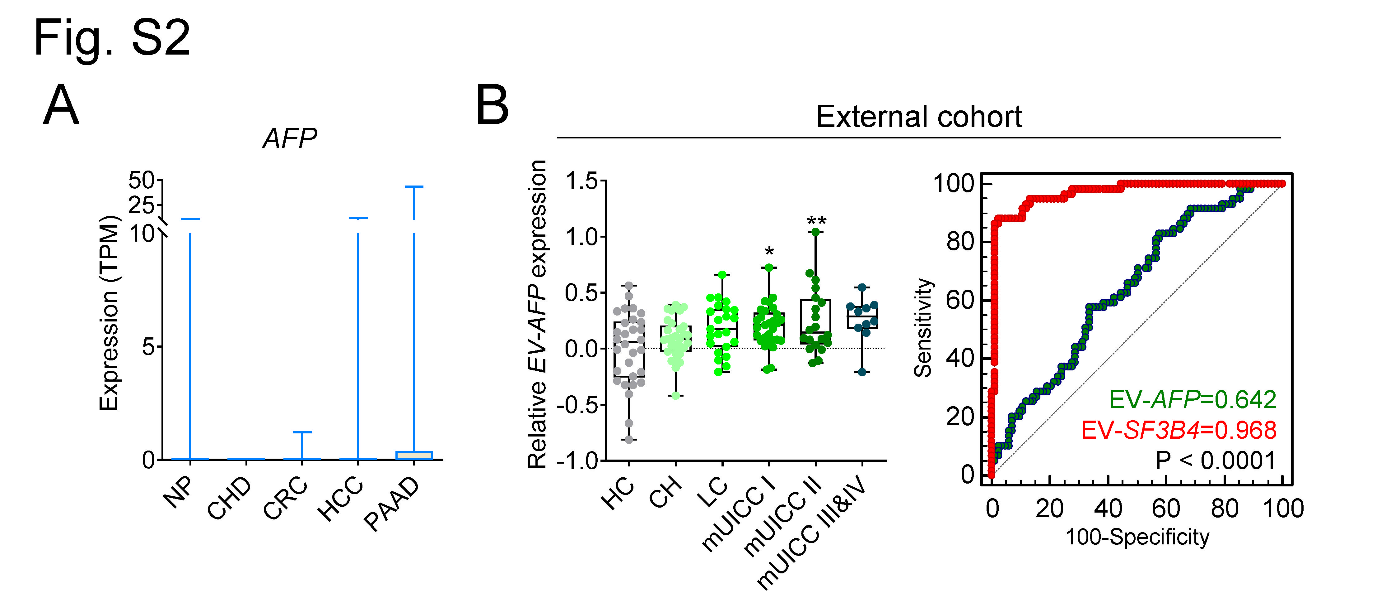


**Supplementary Fig. 2.** EV-*AFP* expression and its diagnostic power in HCC. **A** Box plot of *AFP* expression in blood-derived EVs from different diseases. NP, normal person; CHD, coronary heart disease; CRC, colorectal cancer; HCC, hepatocellular carcinoma; PAAD, pancreatic adenocarcinoma; TPM, transcripts per million **B** Expression of serum EV-*AFP* based on the stage of liver disease (left) and AUCs of serum EV-*AFP* compared to serum EV-*SF3B4* for diagnosing HCC (right) in the external cohort. Statistically significant differences were determined using one-way ANOVA with Tukey’s multiple comparisons test. Compared to HC; **P* <0.05, ***P* <0.01

**
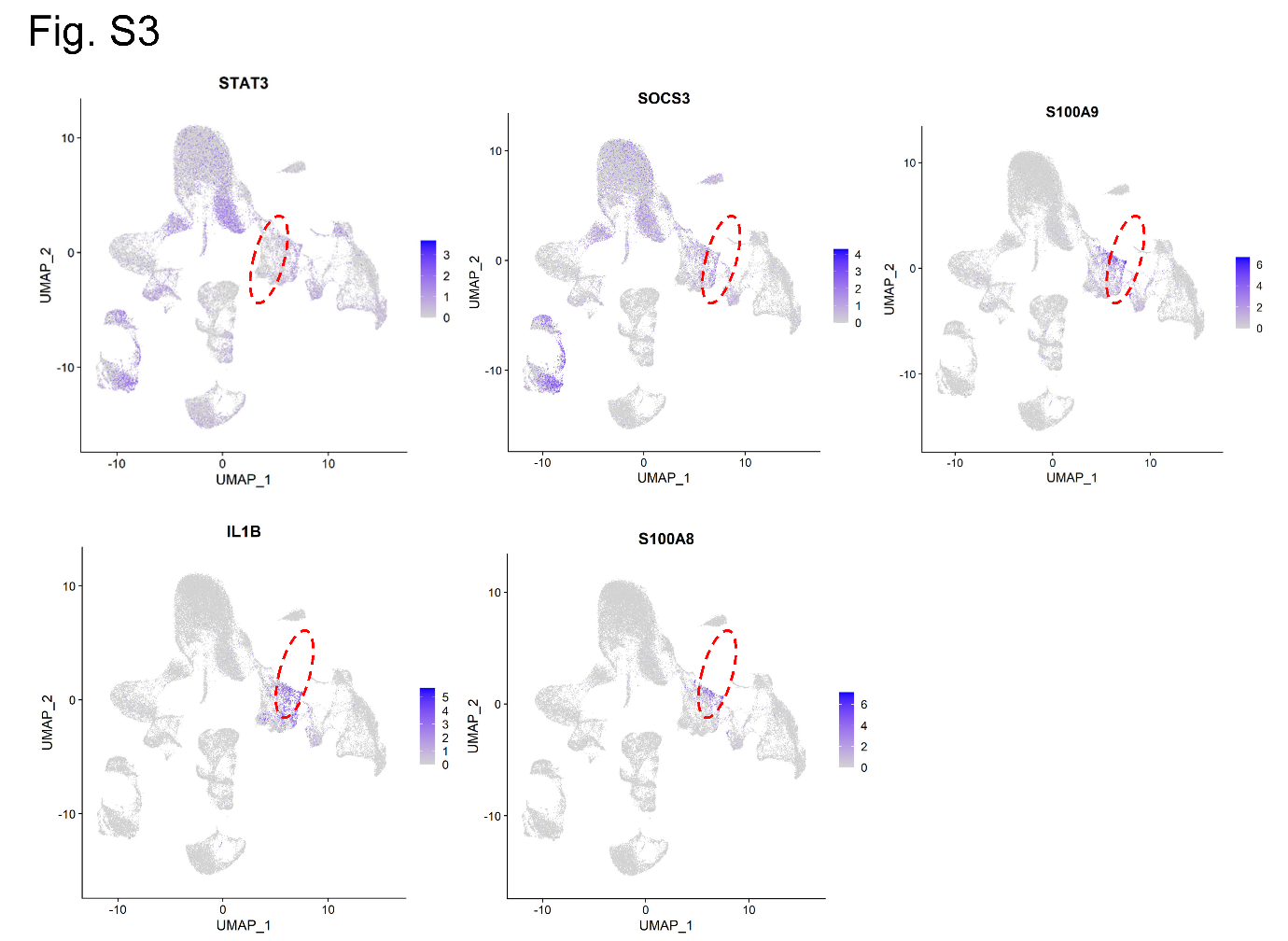
**

**Supplementary Fig. 3.** The distribution of five well-known MDSC markers in whole cells.


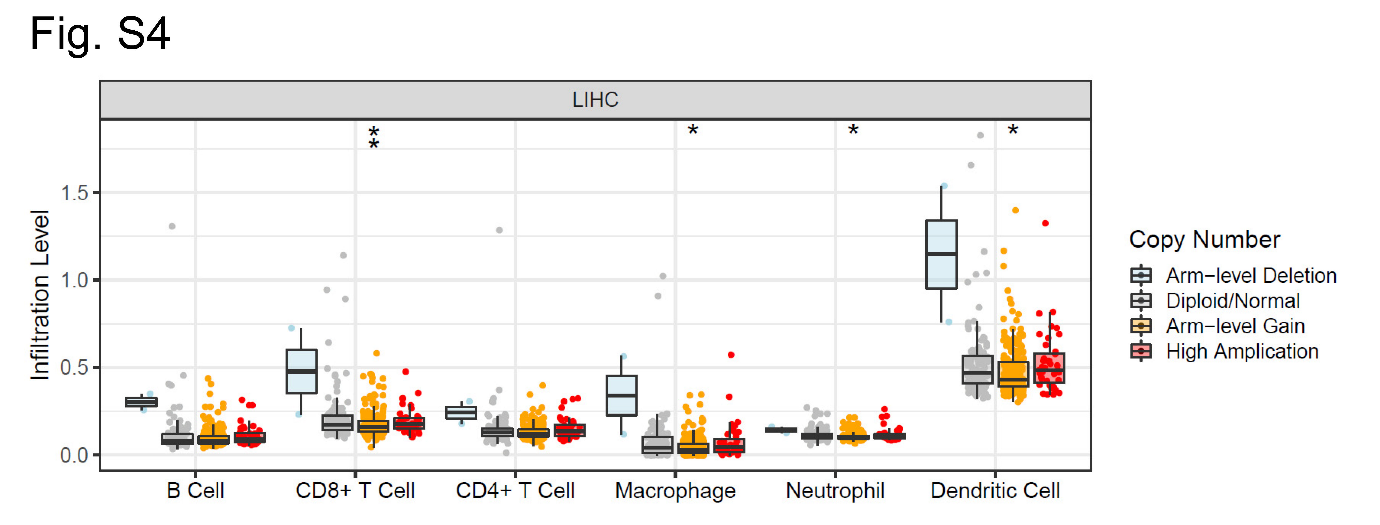


**Supplementary Fig. 4.** The relationship between *SF3B4* Copy Number Variation and immune infiltration. **P* <0.05, ***P* <0.01


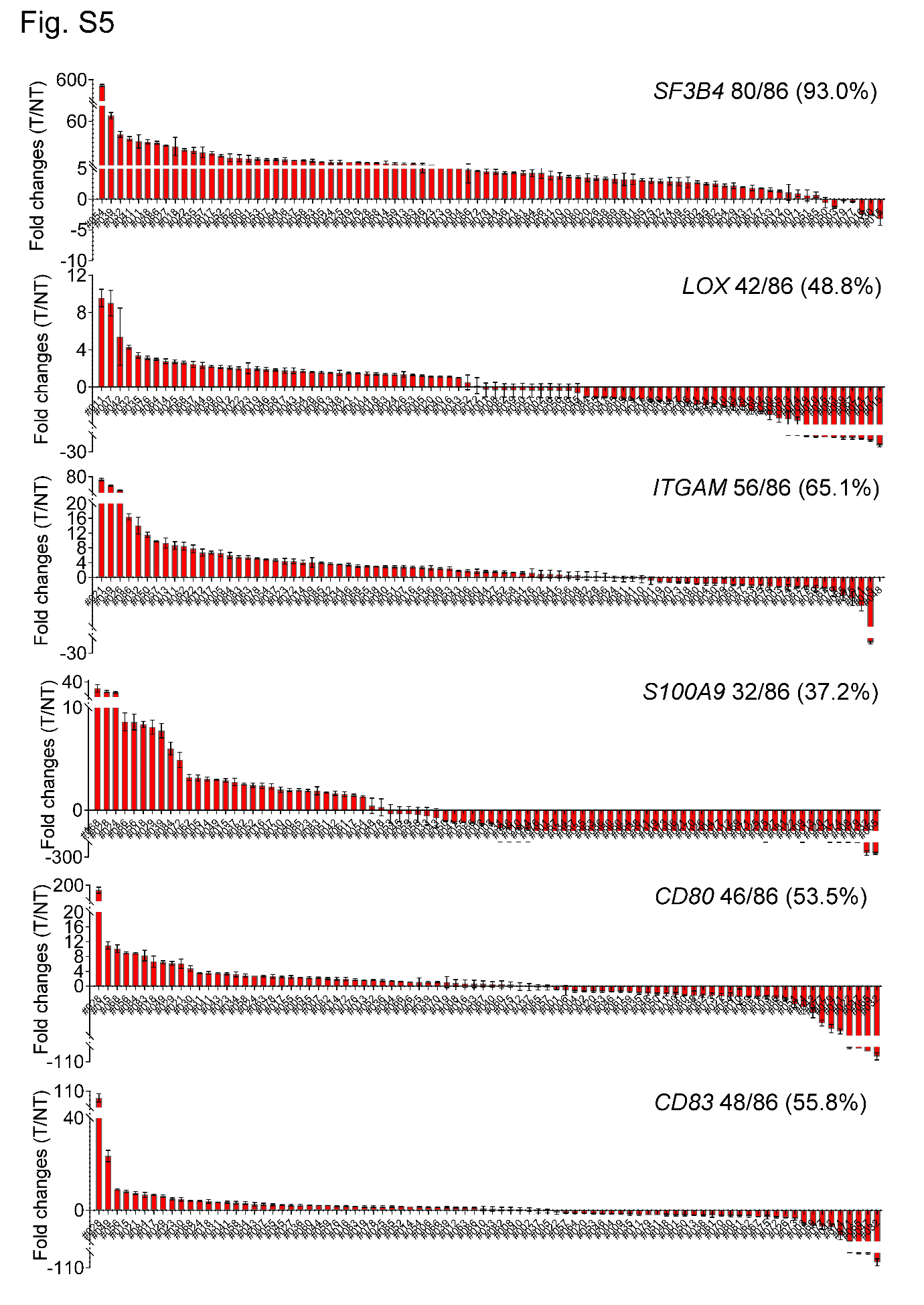


**Supplementary Fig. 5.** Bar chart showing the expression of *SF3B4* and MDSC markers in a set of 86 pairs of HCC tissues.
